# Mathematical modeling of hepatitis C RNA replication, exosome secretion and virus release

**DOI:** 10.1371/journal.pcbi.1008421

**Published:** 2020-11-05

**Authors:** Carolin Zitzmann, Lars Kaderali, Alan S. Perelson

**Affiliations:** 1 University Medicine Greifswald, Institute of Bioinformatics and Center for Functional Genomics of Microbes, Greifswald, Germany; 2 Theoretical Biology and Biophysics, Los Alamos National Laboratory, Los Alamos, New Mexico, United States of America; Yale University, UNITED STATES

## Abstract

Hepatitis C virus (HCV) causes acute hepatitis C and can lead to life-threatening complications if it becomes chronic. The HCV genome is a single plus strand of RNA. Its intracellular replication is a spatiotemporally coordinated process of RNA translation upon cell infection, RNA synthesis within a replication compartment, and virus particle production. While HCV is mainly transmitted via mature infectious virus particles, it has also been suggested that HCV-infected cells can secrete HCV RNA carrying exosomes that can infect cells in a receptor independent manner. In order to gain insight into these two routes of transmission, we developed a series of intracellular HCV replication models that include HCV RNA secretion and/or virus assembly and release. Fitting our models to *in vitro* data, in which cells were infected with HCV, suggests that initially most secreted HCV RNA derives from intracellular cytosolic plus-strand RNA, but subsequently secreted HCV RNA derives equally from the cytoplasm and the replication compartments. Furthermore, our model fits to the data suggest that the rate of virus assembly and release is limited by host cell resources. Including the effects of direct acting antivirals in our models, we found that in spite of decreasing intracellular HCV RNA and extracellular virus concentration, low level HCV RNA secretion may continue as long as intracellular RNA is available. This may possibly explain the presence of detectable levels of plasma HCV RNA at the end of treatment even in patients that ultimately attain a sustained virologic response.

## Author summary

Approximately 70 million people are chronically infected with hepatitis C virus (HCV), which if left untreated may lead to cirrhosis and liver cancer. However, modern drug therapy is highly effective and hepatitis C is the first chronic virus infection that can be cured with short-term therapy in almost all infected individuals. The within-host transmission of HCV occurs mainly via infectious virus particles, but experimental studies suggest that there may be additional receptor-independent cell-to-cell transmission by exosomes that carry the HCV genome. In order to understand the intracellular HCV lifecycle and HCV RNA spread, we developed a series of mathematical models that take both exosomal secretion and viral secretion into account. By fitting these models to *in vitro* data, we found that secretion of both HCV RNA as well as virus probably occurs and that the rate of virus assembly is likely limited by cellular co-factors on which the virus strongly depends for its own replication. Furthermore, our modeling predicted that the parameters governing the processes in the viral lifecycle that are targeted by direct acting antivirals are the most sensitive to perturbations, which may help explain their ability to cure this infection.

## Introduction

Hepatitis C virus (HCV) causes an acute infection that is cleared in some individuals, but which if it becomes chronic can cause liver cirrhosis and hepatocellular carcinoma. Approximately 70 million people worldwide live with chronic hepatitis C, with 400,000 related deaths annually [1]. Hepatitis C can be cured with combinations of direct acting antivirals that inhibit viral replication and which can achieve cure rates above 95% [2]. HCV is a *Hepacivirus* belonging to the family *Flaviviridae* and has a single plus-strand RNA genome. A common feature of all plus-strand RNA viruses including HCV is their ability to rearrange intracellular host membranes to generate so-called replication compartments (RCs) or “replication factories” [3]. In HCV, these RCs derived from the rough endoplasmic reticulum represent a distinct environment for efficient viral genome replication and antiviral immune response protection [4].

Intracellular HCV replication is a controlled spatiotemporal process starting with translation of the viral genome into viral non-structural (NS) and structural proteins, required for HCV genome replication and virus particle formation. The NS proteins form the replicase complex (or replication complex) that is associated with the RC and which is required for viral RNA (vRNA) synthesis. Within the RC the plus-strand RNA genome is replicated into a minus-strand RNA intermediate, which then gives rise to multiple plus-stranded HCV RNA copies. The progeny plus-strand RNA can either undergo another round of RNA synthesis within the RC or be transported out of the RC into the cytoplasm to be translated in order to produce more viral proteins, or together with structural proteins be packaged into virus particles that are secreted from the host cell [4,5].

HCV particle production occurs in association with cytoplasmic lipid droplets (cLDs) that are in close proximity to the endoplasmic reticulum and thus to the RCs. Viral structural proteins and host cellular co-factors are recruited to the cLDs and form together with the viral plus-strand RNA genome virus particles that mature and are released from the cell [3,6]. Due to their limited genome size, viruses depend strongly on cellular co-factors for their own replication. Those host factors are hijacked by the virus and are involved in almost all steps of the viral lifecycle and represent potential drug targets [7,8].

Releasing HCV as enveloped and matured infectious virus particles does not represent the only strategy for viral spread. An infected cell can also secrete vRNA containing exosomes, i.e., small extracellular vesicles [9]. Exosomes are produced from nearly all cell-types with the function of cell-to-cell communication by transferring cellular components, RNAs, and proteins [10]. HCV RNA exosomes and infectious HCV virions are comparable in size (~100 nm for HCV RNA exosomes and 35–100 nm for HCV virions) and density (~1.08 g/ml for HCV RNA exosomes and ~1.10–1.14 g/ml for HCV virions) which makes separation of the particle types difficult [11–13].

Longatti et al. [13] suggest exosomal transfer is a means of HCV transmission between hepatocytes, albeit one that may be less efficient than transmission by true viral particles. These authors showed virion-independent transfer of replication competent HCV RNA in exosomes *in vitro* using an HCV subgenomic replicon lacking viral structural genes including that coding for envelope protein, which suggests that transfer was cell-to-cell. The transfer appeared to require cell-cell contact since coculture experiments done in Transwell plates did not lead to exosomal HCV RNA transfer. Further, concentrated exosomes were not able to directly infect target cells, suggesting that infection by free exosomes is very inefficient compared to infection by authentic viral particles [13]. In comparing the models developed below with data we assume that infectious virus titers measured as focus-forming units is due only to extracellular infectious virions and not exosomes.

In HCV-positive patients and in infected cell cultures, extracellular HCV RNA exosomes have been found in blood plasma and supernatant, respectively [12,14]. These HCV RNA genome carrying exosomes have been shown to generate a “normal” viral HCV infection in HCV-naïve cells by a receptor-independent transmission mechanism [15], although this result remains controversial. Liu et al. [16] found in HCV-positive patient plasma a 3 to 20-fold higher HCV RNA concentration in exosomes compared to exosome-free HCV particles and strongly suggested that HCV infection and transmission occurs as an exosome-associated process. Exosome-associated HCV RNA was found to be infectious and resistant to neutralizing anti-HCV antibodies [16,17]. Furthermore, HCV exosomes carry Ago2, miR-122, and HSP90 proteins that potentially enhance viral replication, as blocking Ago2 or miR-122 leads to suppression of exosomal cell-to-cell transmission [15]. Additionally, HCV exosomes carrying Ago2 and miR-122 trigger macrophage differentiation, which increase inflammation by releasing pro-inflammatory cytokines and collagen, and thereby promotes fibrosis [18,19].

Mathematical modeling of virus-host interactions has proven to be a powerful tool to study viral pathogenesis and transmission as well as antiviral treatment strategies [20–29]. Here we used a previously published intracellular HCV replication model [30] in order to study HCV RNA secretion routes. In that model by Quintela et al. [30] a viral assembly compartment was absent and HCV secretion was modeled simply as a process that leads to loss of intracellular HCV RNA but whether the loss was via exosomes or virus particle production was not specified. Further, the model assumed that HCV RNA secretion occurred from the site of HCV RNA translation, i.e. the cytoplasm, as well as the site of HCV RNA replication. Here we first examine models in which secretion is from the site of translation, the site of replication or as in the Quintela et al. model from both. We then extend this model and make it more biologically realistic by including a separate virion assembly compartment. We fit the set of models we develop to *in vitro* experiments and show that both exosomal secretion and viral production appear to play a role in HCV RNA release from infected cells. Further, the best model fits to the *in vitro* data are obtained when virus assembly/release is limited by host cell resources.

## Methods

### Intracellular HCV replication and HCV RNA secretion models (SM models)

**Fig 1:**
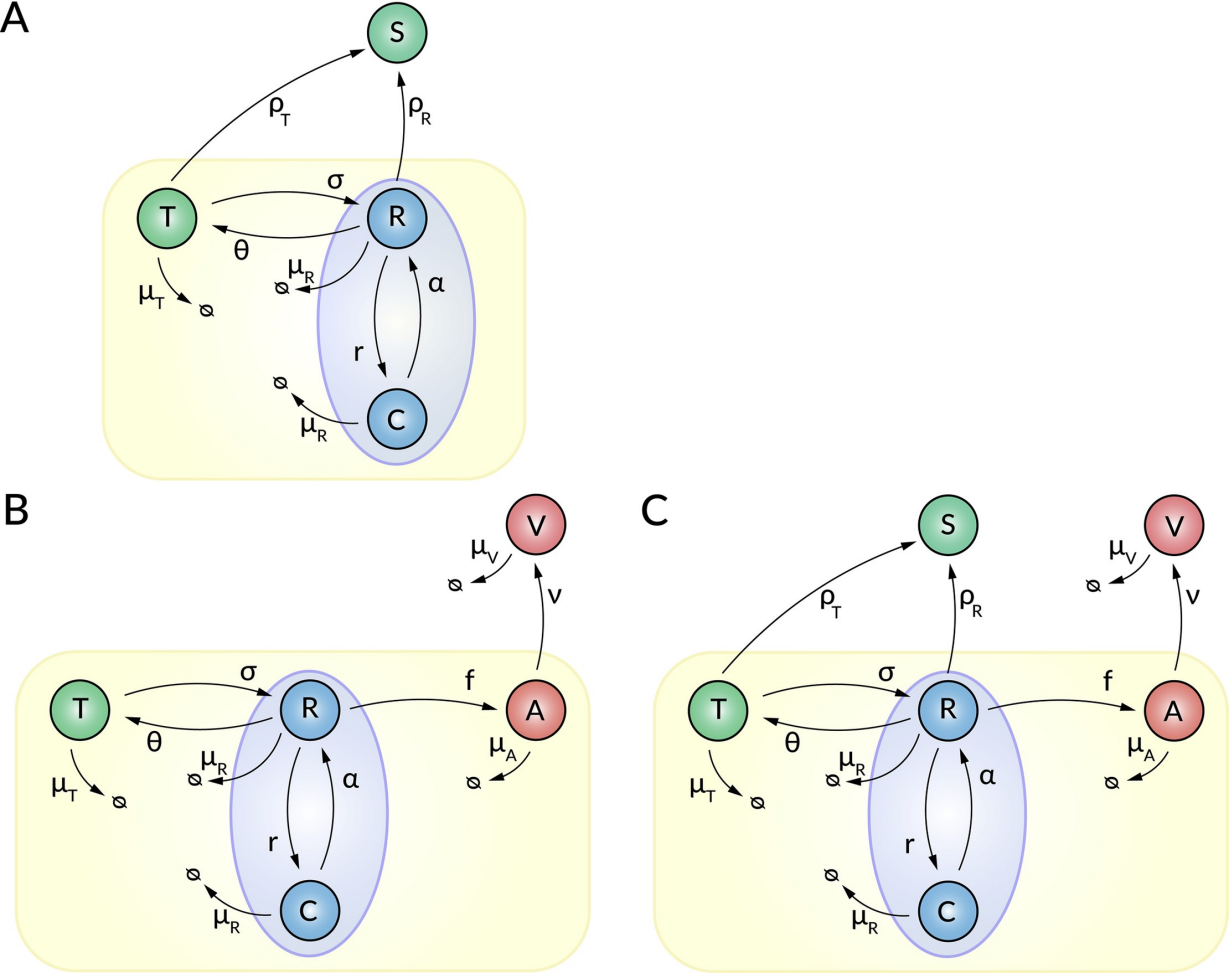
Schematic illustration of the intracellular HCV replication models. (**A**) The hepatitis C RNA secretion model (SM), (**B**) the replication model extended by virus assembly and release without considering RNA secretion (AM), and (**C**) the combined model (CM) accounting for secretion and virus release. Intracellular HCV replication is initiated by plus-strand RNA that is translated (*T*) and transferred into the replication compartment (RC) at rate *σ*. Within the RC, the plus-strand RNA (*R*) is used to synthesize minus-strand RNA (*C*) at maximum rate *r*, which in turn is used for plus-strand RNA synthesis at rate *α*. Plus-strand RNA located at the site of translation (*T*) and/or in proximity to the RC (*R*) may be secreted as HCV RNA containing particles (*S*) at rate *ρ*_*T*_ and/or *ρ*_*R*_, respectively. Newly synthesized plus-strand RNA (*R*) may be transferred to sites of viral assembly (*A*) at rate *f*. HCV particles (*V*) are released from the cell at rate *v* and degrade with rate *μ*_*V*_. Intracellular RNA in the cytoplasm (*T*) degrades with rate *μ*_*T*_, while RNA species (*R* and *C*) within the RC degrade with rate *μ*_*R*_.

The intracellular HCV replication model of [30] contains four different HCV RNA species: plus-strand HCV RNA used for translation (*T*), plus-strand HCV RNA in the RC (*R*) used for replication, minus-strand HCV RNA (*C*) in the RC, which may be in the form of replication complexes, used for replication, and secreted HCV RNA (*S*) (Fig 1A). Here the secretion of intracellular RNA can be due to the RNA being in either exosomes or viral particles or both. We will simply refer to this as secretion of RNA containing particles. The plus-strand RNA in the cytoplasm that can be used for translation, *T*, may be transferred into the RC at rate *σ*. This plus-strand RNA in the RC, *R*, can now be used for the synthesis of minus-strand RNA containing replication complexes, *C*, which in turn can produce more plus-strand RNA, *R*, at rate *α*. As in prior models [30,31], due to limited availability of host factors, the minus-strand RNA synthesis follows a logistic growth law, with maximum rate *r*, which slows as the number of negative strands approaches the RCs carrying capacity *C*_*max*_. Newly synthesized plus-strand RNA, *R*, is transferred back to the site of vRNA translation at rate *θ* in order to produce more viral proteins. The HCV RNA species located at the site of translation degrade with rate *μ*_*T*_. As in Quintela et al. [30], we assume that the HCV RNA species within the RC (*R* and *C*) degrade with the same rate *μ*_*R*_.

Since the secretion route of HCV RNA-containing particles is unknown, we include different pathways in our model: (1) secretion of particles at the site of RNA translation that contain the HCV RNA species, *T*, at rate *ρ_T_*(*t*) (2) the secretion of particles containing newly synthesized HCV RNA in proximity to the RCs, *R*, at rate *ρ_R_*(*t*) and (3) both secretion routes (1) and (2). The mathematical model is described by the following ordinary differential equations (ODEs):

$$\begin{array}{c} \frac{d}{dt}T=\theta R-(\sigma +\rho_{T}(t)+\mu_{T})T,\\ \frac{d}{dt}R=\alpha C+\sigma T-(\theta +\rho_{R}(t)+\mu_{R})R,\\ \frac{d}{dt}C=r(1-\frac{C}{C_{max}})R-\mu_{R}C,\\ \frac{d}{dt}S=\rho_{T}(t)T+\rho_{R}(t)R, \end{array}$$ (1)

with initial conditions reflective of an *in vitro* transfection experiment or high MOI infection experiment in which an average of *T*_0_ positive HCV RNAs are delivered to a cell’s cytoplasm to initiate an infection, i.e. *T*(0) = *T*_0_, *R*(0) = 0, *C*(0) = 0, and *S*(0) = 0. Secretion Model T (SM_T_) refers to the secretion route with *ρ*_*T*_(*t*)≠0 and *ρ*_*R*_(*t*) = 0, Secretion Model R (SM_R_) accounts for the secretion route with *ρ*_*T*_(*t*) = 0 and *ρ*_*R*_(*t*)≠0, while Secretion Model TR (SM_TR_) combines both secretion routes with *ρ*_*T*_(*t*)≠0 and *ρ*_*R*_(*t*)≠0.

In the Quintela et al. [30] model, the secretion of RNA does not begin the instant a cell is infected, but rather it is delayed and then the secretion rate smoothly increases, i.e. ramps up, according to the following function:

$$\rho_{i}(t)=\left\{ \begin{array}{l} 0,t<\tau_{\rho }\\ (1-e^{-k_{\rho }(t-\tau_{\rho })})\rho_{i},\;otherwise \end{array}\right.$$ (2)

with *ρ*_*i*_(*t*)∈{*ρ*_*T*_(*t*),*ρ*_*R*_(*t*)}. We refer to the secretion models that use this delayed ramp-up function as type 1 models and denote them SM_T1_, SM_R1_, and SM_T1R1_. Additionally, we studied the SM_T1R1_ model with equal maximal HCV RNA secretion rates, i.e. *ρ*_*T*_ = *ρ*_*R*_, and secretion delays *τ*_*T*_ = *τ*_*R*_ (SM_T1 = R1_), and a version with individual secretion rates, i.e. *ρ*_*T*_≠*ρ*_*R*_ and *τ*_*T*_≠*τ*_*R*_ (SM_T1≠R1_).

In addition to this function, we also tested secretion models SM_T_, SM_R_, and SM_TR_ that use a simple step-function time delay

$$\rho_{i}(t)=\left\{ \begin{array}{l} 0,t<\tau_{\rho }\\ \rho_{i},\;otherwise \end{array}\right.$$ (3)

rather than the delay given by Eq (2) and refer to those models as type 2 models and denote them SM_T2_, SM_R2_, SM_T2 = R2_, and SM_T2≠R2_, respectively.

Lastly, we explored the possibility that vRNA secretion might be limited by host cellular resources. Thus, we also studied a time delayed release function that after the delay decreases exponentially over time due to host factor limitation

$$\rho_{i}(t)=\left\{ \begin{array}{l} 0,\;t<\tau_{\rho }\\ (e^{-k_{\rho }(t-\tau_{\rho })})\rho_{i},\;otherwise \end{array}\right.$$ (4)

and refer to those models using this function as type 3 models and denote them SM_T3_, SM_R3_, SM_T3 = R3_, and SM_T3≠R3_, (Table 1). Other decreasing functions could also be used but in this initial exploration of the effect of host factor limitation we restrict our analysis to this simple function.

**Table 1:** Secretion model variants. Overview of the different vRNA secretion models (SM) with different secretion routes and time delay functions.

Model	Secretion Route	Time delay function
**SM**_**T1**_	Secretion from the site of translation (*T*) *ρ*_*T*_(*t*)	Delay then ramp-up (Eq 2)
**SM**_**T2**_		Simple delay (Eq 3)
**SM**_**T3**_		Delayed exponential decrease (Eq 4)
**SM**_**R1**_	Secretion from the RC (*R*) *ρ*_*R*_(*t*)	Delay then ramp-up (Eq 2)
**SM**_**R2**_		Simple delay (Eq 3)
**SM**_**R3**_		Delayed exponential decrease (Eq 4)
**SM**_**T1 = R1**_	Equal secretion from sites *T* and *R* *ρ*_*T*_(*t*) = *ρ*_*R*_(*t*) and *τ*_*T*_ = *τ*_*R*_	Delay then ramp-up (Eq 2)
**SM**_**T2 = R2**_		Simple delay (Eq 3)
**SM**_**T3 = R3**_		Delayed exponential decrease (Eq 4)
**SM**_**T1≠R1**_	Individual secretion from sites *T* and *R* *ρ*_*T*_(*t*)≠*ρ*_*R*_(*t*) and *τ*_*T*_≠*τ*_*R*_	Delay then ramp-up (Eq 2)
**SM**_**T2≠R2**_		Simple delay (Eq 3)
**SM**_**T3≠R3**_		Delayed exponential decrease (Eq 4)

### Intracellular HCV replication and virus assembly/release model (AM models)

In order to study HCV RNA secretion in exosomes as distinct from that in virions, we extended the Quintela et al. model (Eq 1) by including an explicit compartment in which the assembly and release of virus occurs in a manner inspired by Benzine et al. [31]. In this model, newly synthesized plus-strand RNA within the RC, *R*, is transferred to sites of virus assembly that are associated with cLDs and assembled at rate *f*(*t*) into intracellular virions, *A*, which are released from the cell and become extracellular virions, *V*, at rate *v* (Fig 1B). Extracellular virus is lost at per capita rate *μ*_*V*_, which in an *in vitro* system is due to degradation or replacement of the culture medium. The intracellular HCV replication model extended by virus assembly and release is given by the following ODEs (extensions in bold):

$$\begin{array}{l} \frac{d}{dt}T=\theta R-(\sigma +\mu_{T})T,\\ \frac{d}{dt}R=\alpha C+\sigma T-(\theta +\mu_{R})R-f(t)R,\\ \;\frac{d}{dt}C=r(1-\frac{C}{C_{max}})R-\mu_{R}C,\\ \;\frac{d}{dt}A=f(t)R-vA,\\ \;\frac{d}{dt}V=vA-\mu_{V}V. \end{array}$$ (5)

with *T*(0) = *T*_0_, *R*(0) = 0, *C*(0) = 0, *S*(0) = 0, *A*(0) = 0, and *V*(0) = 0.

Virus assembly is limited by the availability of structural and non-structural proteins necessary to form the virion as well as the host cell resources involved in virus assembly. Due to these considerations we tested different models for the rate of HCV RNA transport to the assembly site and virion assembly, *f*(*t*). First, a constant rate, i.e., *f*(*t*) = *f* = *const*. Second, a simple time delayed virus assembly rate

$$f(t)=\left\{ \begin{array}{l} 0,\;t<\tau_{f}\\ f,\;otherwise \end{array}\right.$$ (6)

Third, a time-delayed viral assembly rate similar to Eq (2), assuming that after a fixed delay the viral assembly rate increases smoothly until reaching a maximum

$$f(t)=\left\{ \begin{array}{l} 0,\;t<\tau_{f}\\ {(1-e}^{-k_{f}(t-\tau_{f})})f,\;otherwise \end{array}\right.$$ (7)

In analogy with Eq (2), we will call this a delayed ramp-up function.

Fourth, a time-delayed virus assembly rate that then decreases over time due to a limitation or restriction of viral or host cell resources

$$f(t)=\left\{ \begin{array}{l} 0,\;t<\tau_{f}\\ e^{-k_{f}(t-\tau_{f})}f,\;otherwise \end{array}\right.$$ (8)

The various choices for *f*(*t*) that we explore are listed in Table 2.

**Table 2:** Assembly model variants. Overview of the different virion assembly models (AM).

Model	Virus assembly *f*(*t*)
**AM**_**1**_	Constant
**AM**_**2**_	Simple delay (Eq 6)
**AM**_**3**_	Delay then ramp-up (Eq 7)
**AM**_**4**_	Delayed exponential decrease (Eq 8)

### The combined intracellular HCV replication, exosome secretion and virus release model (CM Model)

In order to develop a combined intracellular HCV model, we combined the HCV replication model with the HCV RNA secretion and viral assembly and release models (Fig 1C). This changed the ODEs of Eq (5) to the following with the added exosomal secretion terms indicated in bold:

$$\begin{array}{l} \frac{d}{dt}T=\theta R-(\sigma +\rho_{T}(t)+\mu_{T})T,\\ \frac{d}{dt}R=\alpha C+\sigma T-(\theta +\rho_{R}(t)+\mu_{R})R-f(t)R,\\ \;\frac{d}{dt}C=r(1-\frac{C}{C_{max}})R-\mu_{R}C,\\ \;\frac{d}{dt}S{=\rho }_{T}(t)T+\rho_{R}(t)R,\\ \;\frac{d}{dt}A=f(t)R-vA\\ \;\frac{d}{dt}V=vA-\mu_{V}V. \end{array}$$ (9)

In summary, we study three main models: (A) the HCV RNA secretion model (SM model), (B) the HCV assembly/release model (AM model) and (C) the combination of both, i.e. a combined model (CM model). All models have several sub-models that differ in their time delay functions for HCV RNA secretion (*ρ*_*i*_(*t*)) and virus assembly (*f*(*t*)).

### Fitting the models to data

In order to fit these models to data, global optimization was performed using the Data2Dynamics environment [32] for Matlab 2016b. Parameters were estimated by minimizing the negative of the log likelihood function with the deterministic optimization algorithm *lsqnonlin* and a *Latin hypercube sampling* procedure for generating initial parameter guesses [33]. Uncertainty analysis and the calculation of 95% confidence intervals were performed using the profile likelihood estimation (PLE) routine in Data2Dynamics [32,34].

The models were calibrated using *in vitro* data extracted from Keum et al. [35] using the tool *WebPlotDigitilizer* [36]. Here, we focused on measurements of intracellular HCV plus-strand RNA, intracellular minus-strand RNA, secreted HCV RNA, intracellular cell-associated HCV infectious virus, and released infectious HCV particles [35]. In the Keum et al. experiments, Huh7.5.1 cells were infected with a high multiplicity of infection (MOI = 5) leading to a one-step growth curve where theoretically 99.3% of the cells were infected with at least one HCV particle.

The SM models were fitted to the Keum et al. [35] data shown in their Figs 1A–1C and 2A, where (+)*RNA* = *T*+*R*, (−)*RNA* = *C*, and secreted *RNA* = *S*. The models extended by the viral particle production (AM and CM models) have also been fitted to the cell-associated infectious (intracellular) virus and the extracellular infectious virus data, *inV* and *exV*, respectively, where *inV* = A*f_*inf*_ and *exV* = V*f_*inf*_ and *f*_*inf*_ is the fraction of virus that is infectious and assumed to be the same for intracellular and extracellular virus. Intracellular and extracellular infectious virus measurements were made from the cells and culture media collected from each cell culture well and expressed as focus forming units per well in Fig 2A of Keum et al. [35]. We divided these measurements by the number of cells per well at each time point given in Fig 2D of Keum et al. [35]) in order to change units from focus forming units (FFU) per well to infectious virus particles per cell and infectious particles released per cell. The model combining virus assembly/release and HCV RNA secretion was fitted to the entire data set where secreted *RNA* = *S*+*V* with *S* = *T*+*R*. Thus, in the combined model (CM) secreted RNA comprises HCV RNA secreted from the site of translation, from the RC, and extracellular virus, while intracellular and extracellular infectious virus are *inV* = A*f_*inf*_ and *exV* = V*f_*inf*_. Model selection theory [37,38] based on comparing Akaike Information Criterion (AIC) values was used to find the preferred model.

**Fig 2:**
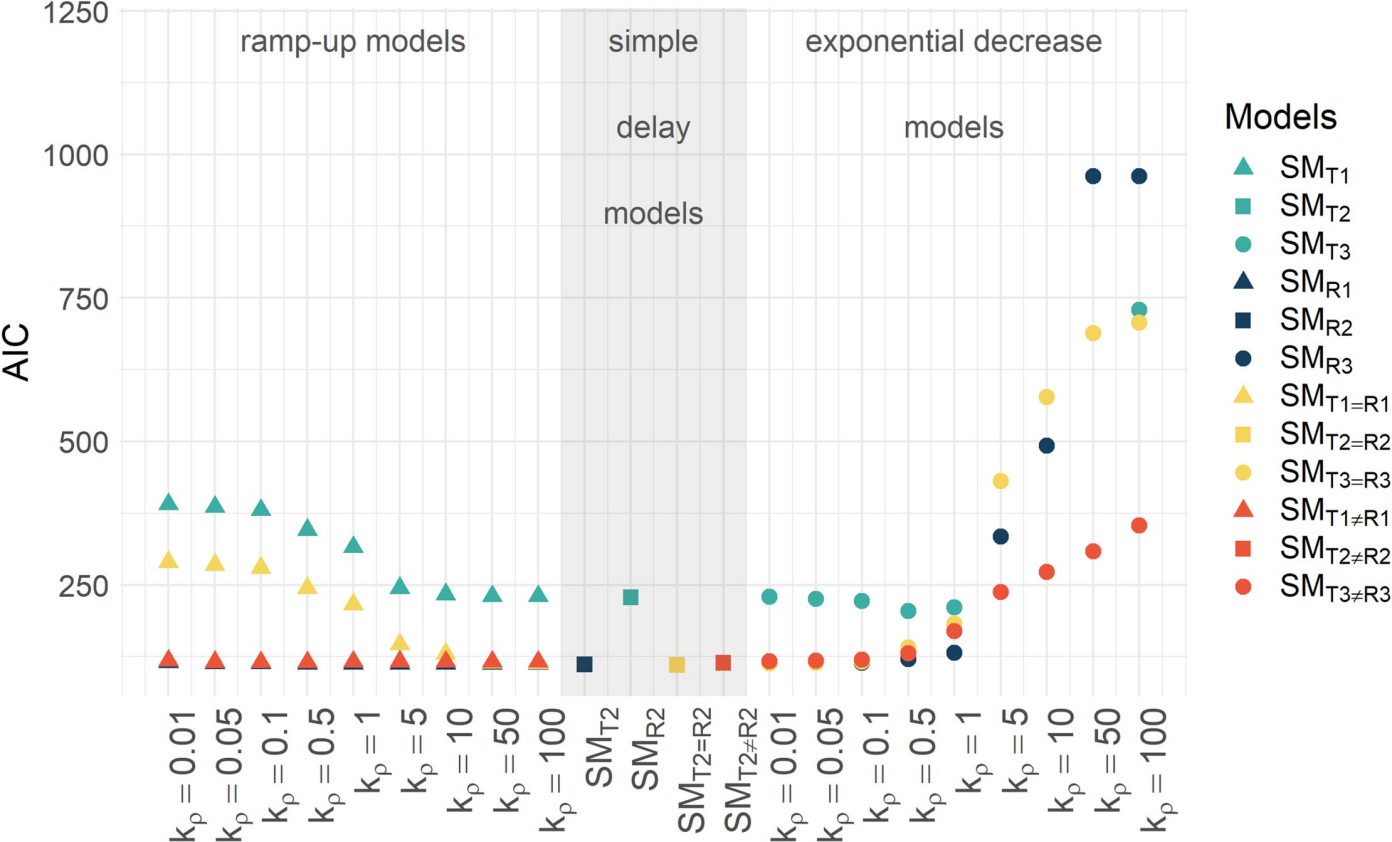
AICs of the HCV RNA secretion models. Best-fit model AICs of the HCV RNA secretion models (Eqs 1 to 4) when the parameter *k*_*ρ*_ determining the rate of ramp-up or limitation was varied (see S1 Data). [▲ = ramp-up models, ■ = simple delay models, ● = exponential decrease models; green = secretion exclusively from site of translation, blue = secretion exclusively from RC, yellow = equal secretion from site of translation and RC (*ρ*_*T*_ = *ρ*_*R*_ and *τ*_*T*_ = *τ*_*R*_), red = individual secretion from site of translation and RC (*ρ*_*T*_≠*ρ*_*R*_ and *τ*_*T*_≠*τ*_*R*_)].

A global parameter sensitivity analysis and the calculation of the first- and total-order sensitivity index was performed using the extended Fourier Amplitude Sensitivity Test (eFAST) package for Matlab 2016b [39]. In brief, the first-order total sensitivity index calculates the sensitivity of a given model parameter *x*_*i*_ on the model output. The total-order sensitivity index is the sum of the first-order sensitivity of a given parameter *x*_*i*_ and the summed sensitivities of the remaining model parameters $\bar{x_{i}}$. The advantage of calculating the total-order sensitivity is that information about parameter interactions are considered, which are not considered by calculating only the first-order sensitivities. Additionally, a dummy parameter has been introduced into the global sensitivity analysis that has no impact on the model output. Hence, model parameters are considered as sensitive, if their total-order sensitivity indices are larger than the total-sensitivity index of this so-called negative control that is ideally zero. For more information see [39].

## Results

### HCV RNA replication and secretion (SM) model

Quintela et al. [30] developed a simple HCV RNA replication and secretion model in which they assumed for simplicity that HCV RNA was secreted from both the cytoplasm and RC at the same rate. Further, they assumed that the secretion was delayed and then ramped-up to a maximum secretion rate as given by Eq (2). Here we revisit the assumptions about the route of secretion and the functional form used to describe the rate of secretion. We evaluated the different possible HCV RNA secretion routes and different functional forms for the rate of secretion by fitting different versions of the model to *in vitro* measurements of plus-strand RNA, minus-strand RNA, and secreted HCV RNA taken from Keum et al. [35]. In all we considered 13 possible models (Tables 1 and 3).

We found that the estimated values of the HCV RNA secretion rates *ρ*_*i*_(*t*),*i* = *R*,*T*, depend strongly on the time delay associated parameters *τ*_*ρ*_ and *k*_*ρ*_. Both parameters led to identifiability problems where small changes in *k*_*ρ*_ hampered the identifiability of the remaining model parameters. Note that a model incorporating a delay followed by a ramp-up function, Eq (2), with large *k*_*ρ*_ becomes equivalent to a model with a simple time delay, Eq (3), as the ramp-up occurs almost instantaneously for large *k*_*ρ*_. Also, if *k*_*ρ*_ is very small, then hardly any ramp-up occurs and hence very little RNA secretion occurs. The same two limits apply for the resource limited model, Eq (4), but with effects of large and small *k*_*ρ*_ reversed. For these reasons, we only studied an intermediate range of fixed *k*_*ρ*_ values with *k*_*ρ*_∈[0.01,100]*d*^−1^ (Fig 2). We found the model SM_T1≠R1_ with secretion from both the site of HCV RNA translation, i.e., the cytoplasm, as well as from the RC, both with compartment specific delayed ramp-up HCV RNA secretion rates was the most likely, i.e., had the lowest negative log likelihood value (-LL), and also was the preferred model, i.e., had the lowest AIC (Table 3).

**Table 3:** Model comparison of the SM models. Negative log likelihood (-LL), AICs, number of estimated model parameters (#P), and $k_{\rho_{i}}$ values of the best-fit models SM_T1_ to SM_TR3_, which were fitted to measurements of plus-strand RNA, minus-strand RNA, and secreted HCV RNA in Keum et al. [35]. The model with the lowest AIC is highlighted in beige and the lowest AIC for each model variant is shown in bold.

Model	Secretion route	Time delay function	#P	$k_{\rho_{i}}$	$\tau_{p_{i}}$	*ρ*_*i*_	-LL	AIC
**SM**_**T1**_	Secretion from the site of translation (*T*)	Delay then ramp-up (Eq 2)	11	*k*_*T*_ = 100 *d*^−1^	*τ*_*T*_ = 0.2 *d*	*ρ*_*T*_ = 1.4 *d*^−1^	91.4	113.4
**SM**_**T2**_		Simple delay (Eq 3)	10	-	*τ*_*T*_ = 0.2 *d*	*ρ*_*T*_ = 1.4 *d*^−1^	**91.4**	**111.4**
**SM**_**T3**_		Delayed exponential decrease (Eq 4)	11	*k*_*T*_ = 0.01 *d*^−1^	*τ*_*T*_ = 0.2 *d*	*ρ*_*T*_ = 1.4 *d*^−1^	91.7	113.7
**SM**_**R1**_	Secretion from the RC (*R*)	Delay then ramp-up (Eq 2)	11	*k*_*R*_ = 100 *d*^−1^	*τ*_*R*_ = 0.01 *d*	*ρ*_*R*_ = 0.05 *d*^−1^	208.6	230.6
**SM**_**R2**_		Simple delay (Eq 3)	10	-	*τ*_*R*_ = 0.01 *d*	*ρ*_*R*_ = 0.05 *d*^−1^	208.5	228.5
**SM**_**R3**_		Delayed exponential decrease (Eq 4)	11	*k*_*R*_ = 0.5 *d*^−1^	*τ*_*R*_ = 0.01 *d*	*ρ*_*R*_ = 0.1 *d*^−1^	**182.5**	**204.5**
**SM**_**T1 = R1**_	Secretion from both sites (*T* and *R*) with *ρ*_*T*_ = *ρ*_*R*_ and *τ*_*T*_ = *τ*_*R*_	Delay then ramp-up (Eq 2)	11	*k*_*T*_ = *k*_*R*_ = 100 *d*^−1^	*τ*_*T*_ = *τ*_*R*_ = 0.01 *d*	*ρ*_*T*_ = *ρ*_*R*_ = 0.03 *d*^−1^	91.5	113.5
**SM**_**T2 = R2**_		Simple delay (Eq 3)	10	-	*τ*_*T*_ = *τ*_*R*_ = 0.01 *d*	*ρ*_*T*_ = *ρ*_*R*_ = 0.03 *d*^−1^	**90.7**	**110.7**
**SM**_**T3 = R3**_		Delayed exponential decrease (Eq 4)	11	*k*_*T*_ = *k*_*R*_ = 0.01 *d*^−1^	*τ*_*T*_ = *τ*_*R*_ = 0.01 *d*	*ρ*_*T*_ = *ρ*_*R*_ = 0.03 *d*^−1^	91.0	113.0
**SM**_**T1≠R1**_	Secretion from both sites (*T* and *R*) with *ρ*_*T*_≠*ρ*_*R*_ and *τ*_*T*_≠*τ*_*R*_	Delay then ramp-up (Eq 2)	13	*k*_*T*_ = *k*_*R*_ = 100 *d*^−1^	*τ*_*T*_ = 0.01 *d* *τ*_*R*_ = 0.4 *d*	*ρ*_*T*_ = 0.04 *d*^−1^ *ρ*_*R*_ = 0.03 *d*^−1^	90.4	116.4
**SM**_**T2≠R2**_		Simple delay (Eq 3)	12	-	*τ*_*T*_ = 0.02 *d* *τ*_*R*_ = 0.4 *d*	*ρ*_*T*_ = 0.04 *d*^−1^ *ρ*_*R*_ = 0.03 *d*^−1^	**90.4**	**114.4**
**SM**_**T3≠R3**_		Delayed exponential decrease (Eq 4)	13	*k*_*T*_ = *k*_*R*_ = 0.01 *d*^−1^	*τ*_*T*_ = 0.2 *d* *τ*_*R*_ = 0.4 *d*	*ρ*_*T*_ = 0.05 *d*^−1^ *ρ*_*R*_ = 0.03 *d*^−1^	90.8	116.8
**SM**_**T1≠R1**_	(+)RNA secretion from the RC (*R*) and the site of translation (*T*) with *ρ*_*T*_≠*ρ*_*R*_ and *τ*_*T*_≠*τ*_*R*_ and *k*_*T*_≠*k*_*R*_	Delay then ramp-up (Eq 2)	14	*k*_*T*_ = 50 *d*^−1^ *k*_*R*_ = 5 *d*^−1^	*τ*_*T*_ = 0.1 *d* *τ*_*R*_ = 3 *d*	*ρ*_*T*_ = 0.8 *d*^−1^ *ρ*_*R*_ = 0.05 *d*^−1^	**81.6**	**109.6**

**Fig 3:**
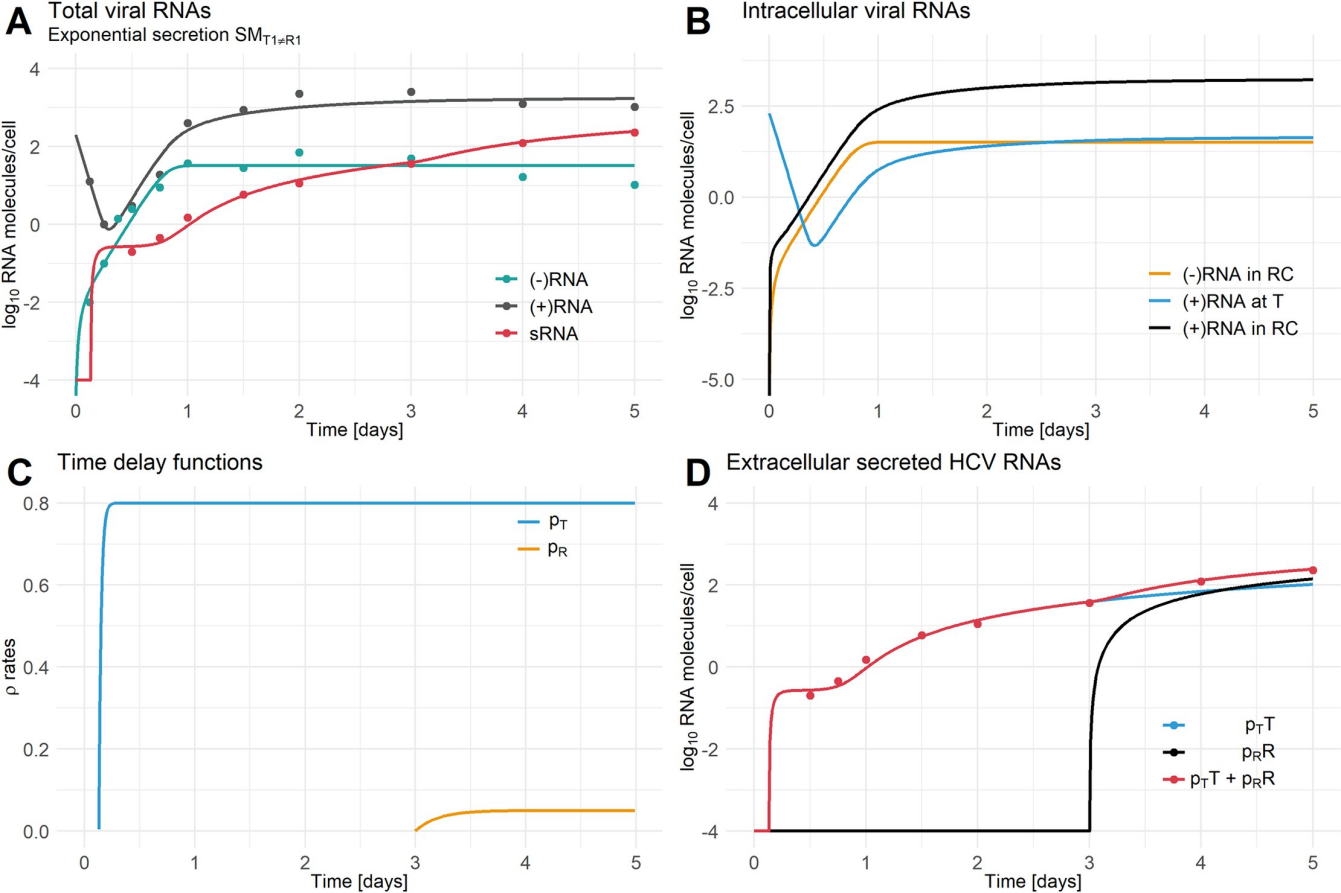
Individual HCV secretion from the site of translation and the replication compartment. A) Best-fit secretion model which includes independent HCV RNA secretion from the site of translation and the RC (SM_T1≠R1_ with *τ*_*T*_ = 0.1 *d*, *τ*_*R*_ = 3 *d*, *ρ*_*T*_ = 0.8 *d*^−1^, *ρ*_*R*_ = 0.05 *d*^−1^, *k*_*T*_ = 50 *d*^−1^, and *k*_*R*_ = 5 *d*^−1^). B) Intracellular HCV RNA species at the site of translation (*T*) and in the RC (*R*). C) Time delay functions for the HCV RNA secretion with *ρ*_*T*_ = (+)RNA secretion rate from site of translation and *ρ*_*R*_ = (+)RNA secretion rate from the RC. D) Composition of extracellular secreted HCV RNA with *ρ*_*T*_*T* denoting the amount of extracellular secreted (+)RNA from the site of translation, *ρ*_*R*_*R* denoting the amount of extracellular secreted (+)RNA from the RC and *ρ*_*T*_*T+ρ*_*R*_*R* denoting the sum of all extracellular secreted HCV (+)RNA species (sRNA). [(-)RNA = minus-stranded RNA, (+)RNA = plus-stranded RNA, sRNA = secreted HCV RNA (see S1 Data)]. Data has been taken from [35] Fig 1A–1C. See Table 4 for parameter information.

The different best-fit HCV RNA secretion models for each secretion route generate a different shape for the HCV RNA secretion curve versus time, while the models accounting for the same HCV RNA secretion route generate the same shape HCV RNA release curve (S1 and S2 Figs). The models assuming HCV RNA secretion from the site of translation (SM_Ti_, SM_Ti = Ri_, SM_Ti≠Ri_ with i = 1,2,3) showed a very rapid initiation of HCV RNA secretion followed by a slower increase and represented the models with the lowest AIC (Fig 3A–3C, S1 and S2 Tables), while the models that only considered HCV RNA secretion from the RC (SM_Ri_ with i = 1,2,3) showed a smooth increase in extracellular HCV and did not fit the early secreted RNA data well (S1 and S2 Figs). Hence, models including RNA secretion at the site of translation were highly preferred compared to models in which the secretion of HCV RNA was exclusively from the RC. Moreover, the majority of HCV RNA secreted from the cell was associated with HCV RNA at the site of translation. More specifically, 57% of the secreted HCV RNA came from the site of translation and 43% from the RC when summed over the 5 day experiment. Initially, HCV RNA is secreted exclusively from the site of translation starting about 3 hours post-infection (hpi). After 3 days, HCV RNA starts being secreted from the RC and on average over the period from days 3 to 5 secretion from the site of translation and the RCs contribute equally to the HCV RNA secretion (Fig 3D).

**Table 4:** Parameter values and 95% confidence intervals of the best-fit models SM_T1≠R1_, AM_4_, and CM_4_. Note that parameter values marked with * were fixed due to previous assumptions (time delay parameters: $k_{f},\;k_{\rho_{T}},\;k_{\rho_{R}}$), fixed after sensitivity/identifiability analysis (*μ*_*R*_) and personal communication (*μ*_*V*_) (see Methods). The degradation rate of HCV RNA within the RC was set to *μ*_*R*_ = 0 as was the rate of degradation of intracellular virus *μ*_*A*_ = 0 (see main text). For parameter identifiability profiles see S3, S4 and S5 Figs.

Parameter	Description	SM_T1≠R1_	AM_4_	CM_4_	Unit
***AIC***		109.6	214.0	198.1	
***ρ***_***T***_	*S* secretion rate	0.8 [0.4, 2.0]	-	0.09 [0.05, 0.2]	*d*^−1^
***ρ***_***R***_	*S* secretion rate	0.05 [0.01, 0.09]	-	0.09 [0.06, 0.1]	*d*^−1^
$\tau_{\rho_{T}}$	*S* secretion delay	0.13 [0.09, 0.18]	-	0.01 [0.01, 0.05]	*d*
$\tau_{\rho_{R}}$	*S* secretion delay	3 [2.4, 3]	-	2.3 [2, 2.5]	*d*
$k_{\rho_{T}}$	*S* secretion rate parameter	50 *	-	10 *	*d*^−1^
$k_{\rho_{R}}$	*S* secretion rate parameter	5 *	-	1 *	*d*^−1^
*f*	*V* assembly rate	-	2.7 [2.2, 8.3]	0.63 [0.58, 0.7]	*d*^−1^
***τ***_***f***_	*V* assembly delay	-	0.48 [0.46, 0.49]	0.48 [0.46, 0.49]	*d*
***k***_***f***_	*V* assembly rate parameter	-	2 *	2 *	*d*^−1^
***T***_**0**_	Initial number of HCV RNAs	202 [66, 250]	196 [160, 262]	236 [88, 588]	molecules/cell
***C***_***max***_	Maximal number of *C*	32.5 [27.2, 38.8]	35.1 [30.6, 40.8]	32.7 [27.6, 38.6]	molecules/cell
***σ***	Rate of transfer of *T* to the RC	0.007 [0.003, 0.02]	0.006 [0.005, 0.009]	0.006 [0.002, 0.01]	*d*^−1^
***θ***	Rate of transfer of *R* to the cytoplasm	0.6 [0.3, 1.0]	0.9 [0.6, 1.2]	0.7 [0.5, 1.0]	*d*^−1^
***r***	*C* replication rate	3.5 [2.5, 5.1]	3.5 [3.1, 3.9]	3.6 [2.8, 4.6]	*d*^−1^
***α***	*R* replication rate	34.8 [26.5, 45.9]	38.6 [34.9, 42.5]	35.6 [27.7, 45.9]	*d*^−1^
***ν***	*V* release rate	-	0.86 [0.74, 0.97]	0.85 [0.76, 0.97]	
***μ***_***T***_	Cytoplasmic RNA degradation rate	22.3 [16.1, 25.8]	22.2 [20.2, 24.5]	23.1 [17.5, 30.0]	*d*^−1^
***μ***_***R***_	*R* and *C* degradation rates	0	0	0	*d*^−1^
***μ***_***A***_	*A* degradation rate	0	0	0	*d*^−1^
***μ***_***V***_	*V* degradation rate	-	2.77 *	2.77 *	*d*^−1^
***f***_***inf***_	Fraction of *A* and *V* that is infectious	-	0.0014 [0.0009, 0.0018]	0.0055 [0.0048, 0.0063]	

Lastly, we examined the possibility that minus-strand RNA or double stranded RNA containing a minus-strand might also be secreted (S1 Text and S6 Fig) since double stranded HCV RNA has been seen in exosomes [40]. We found the best model with minus-strand secretion (lowest AIC, S7, S8 and S9 Figs) had plus-strand RNA secretion from the sites of translation and replication and minus-strand RNA secretion from the RC (SM_T1≠R1≠C1_, S3 Table). The models including minus-strand RNA secretion showed lower AICs compared to the models excluding minus-strand RNA (S3 and S4 Tables). However, we have not pursued this model further as we have no quantitative measurements of minus-strand secretion to fit the model to and validate these results.

### HCV replication model with virion assembly and release (AM)

Quintela et al. [30] implicitly assumed that secreted HCV RNA was packaged into virions but did not explicitly model virion assembly. Benzine et al. [31] introduced a model that had an explicit virion assembly compartment, which is truer to the biology as virion assembly is known to occur in association with cLD [41]. Inspired by the Benzine et al. model, we explicitly modeled virus particle production. To do this, we modified the HCV replication and secretion model by eliminating HCV RNA secretion and replacing it by virus assembly and release (Eq 5). Thus, this model is more akin to the standard viral dynamics model that only considers viral production as a source of extracellular HCV RNA [21,42]. In the context of this model, we examined four different functions for the rate of virus assembly [*f*(*t*)]: (i) a constant viral assembly rate, (ii) a simple time delayed virus assembly rate (Eq 6), (iii) a delayed ramp-up virus assembly rate (Eq 7), and (iv) a delayed decrease in the viral assembly rate, which we interpret as indicating a limitation in viral or host cell resources (Eq 8). For each different function *f*(*t*), we evaluated the HCV assembly and release models (AM models) without accounting for HCV RNA secretion via exosomes (Table 2).

**Fig 4:**
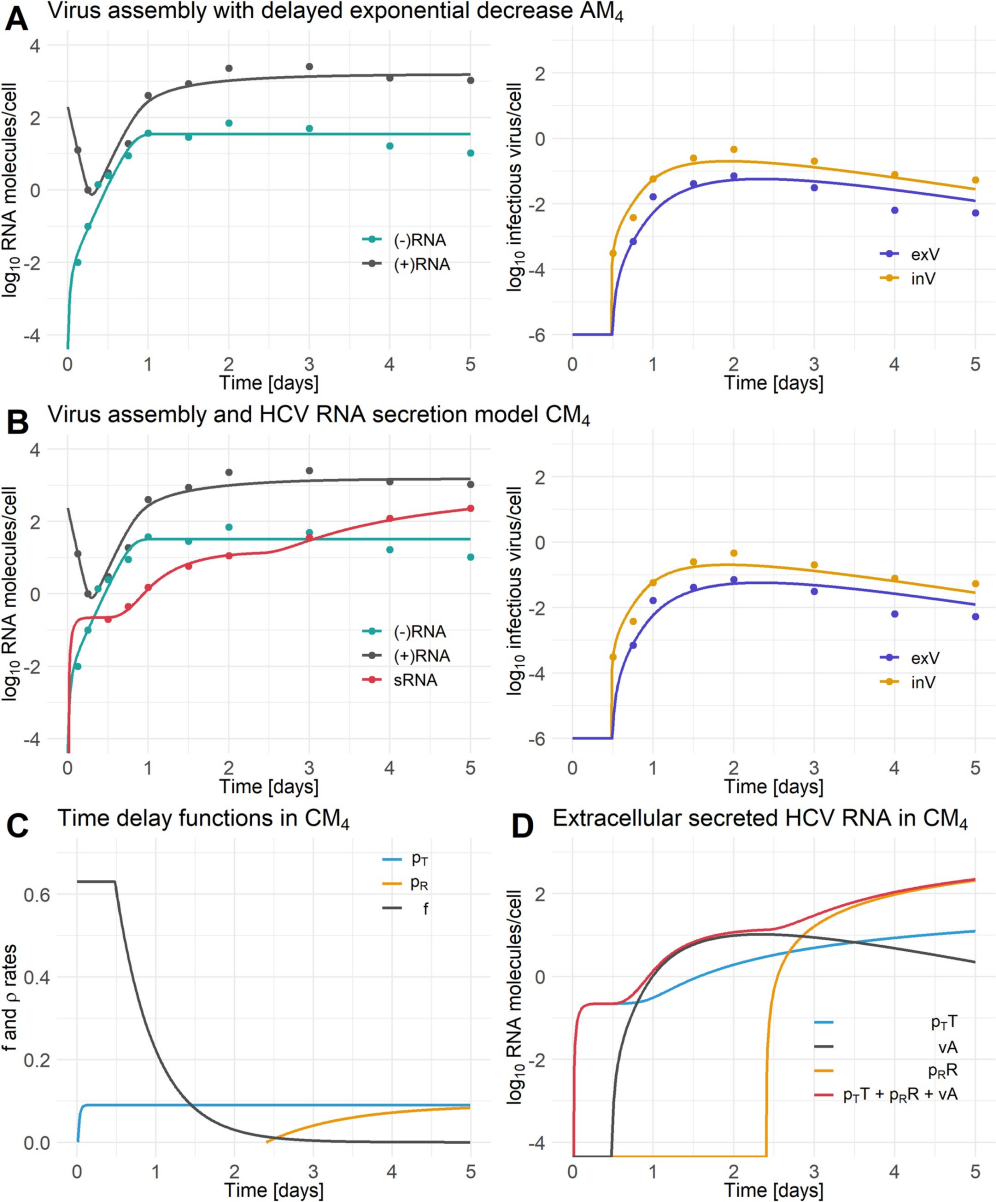
Best fits of the AM_4_ and CM_4_ models. A) Best-fit of the HCV assembly and release models AM_4_ (Eqs 4 to 9). B) Best fit of the combined HCV model CM (Eq 9) with individual HCV RNA secretion (*ρ*_*T*_≠*ρ*_*R*_, *τ*_*T*_≠*τ*_*R*_, and *k*_*T*_≠*k*_*R*_). C) Time delay functions of the combined model (CM) for virus assembly rate and HCV RNA secretion rates with *ρ*_*T*_ = (+)RNA secretion rate from site of translation, *ρ*_*R*_ = (+)RNA secretion rate from the RC and *f* = virus assembly rate. D) Extracellular secreted HCV RNA in the combined model (CM) with *ρ*_*T*_*T* denoting the amount of extracellular secreted (+)RNA from the site of translation, *ρ*_*R*_*R* denoting the amount of extracellular secreted (+)RNA from the RC, *ρ*_*T*_*T+ρ*_*R*_*R* denoting the sum of all extracellular secreted HCV (+)RNA species (sRNA), and *ρ*_*T*_*T+ρ*_*R*_*R+νA* denoting the sum of all extracellular secreted HCV (+)RNA species and virus (exV). [(-)RNA = minus-stranded RNA, (+)RNA = plus-stranded RNA, sRNA = secreted HCV RNA, exV = extracellular infectious virus, inV = intracellular infectious virus in the assembly compartment (see S1 Data)]. Data has been taken from [35] Figs 1A–1C and 2A. The data in Fig 2A of Keum et al. was given per well. We divided the measured data by the number of cells per well (Fig 2D Keum et al. [35]) to give the infectious intracellular and extracellular virus released per cell. See for parameter information Table 4.

The extracellular infectious virus loss rate was set to *μ*_*V*_ = 2.77 *d*^−1^, which corresponds to an infectivity half-life of 6 *h*, a value measured at 37° C in *in vitro* experiments (S. Uprichard, personal communication). However, similar to the HCV RNA secretion model, trying to estimate the continuous time delay associated parameter, *k*_*f*_, for the function describing the rate of virus assembly, *f*(*t*), led to identifiability problems and hampered the calculation of 95% confidence intervals of the estimated model parameters. Therefore, we performed various model fits with fixed values of *k*_*f*_∈[0.1, 10] *d*^−1^. From the identifiability analysis of the AM models using likelihood profiling, we found that the degradation rates of RNA within the RC, *μ*_*R*_, could not be uniquely identified. When we set a lower bound on the parameter value, fitting would generate an estimate at this bound. Trying even lower bounds did not improve this and thus we decided to set *μ*_*R*_ = 0 consistent with the observation that the replication compartment provides protection against nuclease mediated degradation of HCV RNA [43]. Note that we also neglected intracellular virus degradation, since we estimated a very small value for the intracellular virus decay (*μ*_*A*_<0.001 *d*^−1^) that was in agreement with the expectation that intracellular virus is protected from degradation within the assembly compartment. Hence, we set *μ*_*A*_ = 0. The remaining model parameters were estimated by fitting each model to the measurements of plus-strand RNA, minus-strand RNA, intracellular and extracellular infectious virus from Keum et al. [35]. Note that we introduced a parameter, *f*_*inf*_, that scales the assembled (intracellular) and released (extracellular) virus to infectious virus as infectious virus was measured in Keum et al. [35]. We found that the model incorporating a delayed limitation in the viral assembly process (Eq 8) was the best model as it had the lowest AIC (AM_4_) (Fig 4, Table 5).

**Table 5:** Model comparison of the AM models. Negative log likelihood (-LL), AICs and number of estimated model parameters (#P) of the assembly models (AM) without exosome secretion. AM_1_ to AM_4_ were each fitted to plus-strand RNA, minus-strand RNA, intracellular and extracellular virus from Keum et al. [35]. The model with the lowest AIC is highlighted in beige.

Model	*f*(*t*)	# P	*k*_*f*_	*τ*_*f*_	-LL	AIC
**AM**_**1**_	Virus assembly (*f*(*t*)) const.	12	-	-	443.1	467.1
**AM**_**2**_	Virus assembly (*f*(*t*)) simple delay (Eq 6)	13	-	0.2 *d*	436.6	462.6
**AM**_**3**_	Virus assembly (*f*(*t*)) delayed then ramp-up (Eq 7)	14	0.1 *d*^−1^	0.4 *d*	317.8	345.8
**AM**_**4**_	Virus assembly (*f*(*t*)) delayed exponential decrease (Eq 8)	14	2.0 *d*^−1^	0.35 *d*	**186.0**	**214.0**

### An HCV replication model with both virion assembly and release and exosome secretion: the combined model (CM)

As experimental evidence suggests, HCV infected cells produce virions as well as secrete HCV RNA containing exosomes [15], we combined the HCV replication model with the HCV RNA secretion and virus assembly and release model (Eq 9). Here, we built upon our previous best model fits of the HCV RNA secretion (SM) and the HCV assembly and release model (AM). Since we found the preferred model (SM) included HCV RNA secretion via *T* and *R* (SM_T1≠R1_ with $k_{\rho_{T}}\neq k_{\rho_{R}}$), we chose to allow both HCV RNA secretion pathways for the combined model. In order to study, whether our assumption of a limited virus assembly and release is still valid or if HCV RNA secretion via exosomes has an impact on the rate of virus assembly and release, we again tested the four possible virus assembly rate functions in this CM model with HCV RNA secretion: CM_1_ with a const. virus assembly rate, CM_2_ with a simple delayed virus assembly rate (Eq 6), CM_3_ with a delayed virus assembly rate that ramps-up (Eq 7), and CM_4_ with a delayed virus assembly rate that decreases exponentially over time (Eq 8) (Table 6).

**Table 6:** Model comparison of the CM models. Negative log likelihood (-LL), AICs and number of estimated model parameters (#P) of the combined model (CM) with exosome secretion and virus assembly and release. CM_1_ to CM_4_ were each fitted to plus-strand RNA, minus-strand RNA, secreted HCV RNA, intracellular and extracellular virus from Keum et al. [35]. The model with the lowest AIC is highlighted in beige.

Model	*f*(*t*)	# P	$k_{\rho_{i}}$	$\tau_{\rho_{i}}$	*ρ*_*i*_	*k*_*f*_	*τ*_*f*_	*f*	-LL	AIC
**CM**_**1**_	Virus assembly (*f*(*t*)) const.	18	$k_{\rho_{T}}=50\;d^{-1\;}k_{\rho_{R}}=1d^{-1}$	$\tau_{\rho_{T}}=0.3\;d$ $\tau_{\rho_{R}}=2.5\;d$	*ρ*_*T*_ = 1000 *d*^−1^ *ρ*_*R*_ = 0.2 *d*^−1^	-	-	0.0001 *d*^−1^	571.7	607.7
**CM**_**2**_	Virus assembly (*f*(*t*)) simple delay (Eq 6)	19	$k_{\rho_{T}}=50\;d^{-1}\;k_{\rho_{R}}=1\;d^{-1}$	$\tau_{\rho_{T}}=0.3\;d$ $\tau_{\rho_{R}}=2.5\;d$	*ρ*_*T*_ = 1000 *d*^−1^ *ρ*_*R*_ = 0.15 *d*^−1^	-	0.01 *d*	0.0001 *d*^−1^	571.7	609.7
**CM**_**3**_	Virus assembly (*f*(*t*)) delayed then ramp-up (Eq 7)	20	$k_{\rho_{T}}=50\;d^{-1}\;k_{\rho_{R}}=1\;d^{-1}$	$\tau_{\rho_{T}}=0.3\;d$ $\tau_{\rho_{R}}=2.5\;d$	*ρ*_*T*_ = 1000 *d*^−1^ *ρ*_*R*_ = 0.19 *d*^−1^	2 *d*^−1^	0.01 *d*	0.02 *d*^−1^	614.6	654.6
**CM**_**4**_	Virus assembly (*f*(*t*)) delayed exponential decrease (Eq 8)	20	$k_{\rho_{T}}=10\;d^{-1}$ $k_{\rho_{R}}=1\;d^{-1}$	$\tau_{\rho_{T}}=0.01\;d$ $\tau_{\rho_{R}}=2.35\;d$	*ρ*_*T*_ = 0.09 *d*^−1^ *ρ*_*R*_ = 0.09 *d*^−1^	2 *d*^−1^	0.5 *d*	0.7 *d*^−1^	**158.1**	**198.1**

The combined models (CM_1_, CM_2_, CM_3_, and CM_4_) were fitted to measurements of plus-strand RNA, minus-strand RNA, secreted HCV RNA, intracellular, and extracellular infectious virus from Keum et al. [35]. Similar to the HCV assembly and release model, we fixed the loss rate for extracellular infectious virus to *μ*_*V*_ = 2.77 *d*^−1^, set the degradation rates *μ*_*R*_ = *μ*_*A*_ = 0 and performed independent model fits scanning through fixed values of *k*_*f*_∈[0.1, 10] *d*^−1^ and $k_{\rho_{i}}\in [0.01,100]\;d^{-1}$ and then utilized the values that gave the lowest AIC (Table 6). The remaining model parameters were estimated (Table 4).

The model that takes into account a delayed decrease in the virus assembly and release rate (CM_4_) was able to fit the five types of longitudinal measurements made by Keum et al. [35] and showed a substantially lower AIC than the other three CM models (Table 6). Furthermore, model CM_4_ had a lower AIC than the model neglecting HCV RNA secretion, i.e., model AM_4_ (Table 4). However, despite allowing HCV RNA to be secreted, the model could still accurately fit the total intracellular plus-strand RNA and showed a comparable dynamic to the plus-strand RNA in model AM_4_ that neglects HCV RNA secretion (Fig 4A and 4B). Similar to the HCV RNA secretion model (SM_T1≠R1_), the HCV RNA secretion process of the combined model (CM_4_) showed immediate HCV RNA secretion from the site of translation, while RNA secretion from the RC was 2.3 days delayed and contributes the majority of the extracellular secreted HCV RNA amount (Fig 4C and 4D). After around 10 hrs. virus starts being secreted from the cell, reaches a peak at 2 days post-infection (dpi) and falls slightly subsequently (Fig 4B, right panel).

### Sensitivity analysis

In order to gain insight into the impact of model parameters on the combined model, we performed a global sensitivity analysis for all HCV species (plus-strand RNA, minus-strand RNA, secreted RNA, intracellular and extracellular infectious virus) for two different time points, day 1 and day 3 post-infection (see S2 Text and S10 Fig). The amount of secreted HCV RNA was highly sensitive to the rate of cytoplasmic RNA degradation, *μ*_*T*_, and to the parameters describing the secretion process from the site of translation, *τ*_*ρT*_ and *k*_*ρT*_, at 1 dpi as well as the virus replication and transfer parameters, *r*,*α*,*τ* and *C*_*max*_ at 3 dpi. The amount of intracellular infectious virus at 3 dpi was sensitive to the secretion process parameters, while the amounts of both infectious virus species at 3 dpi were sensitive to the virus assembly and release parameters.

### Antiviral drug treatment

**Fig 5:**
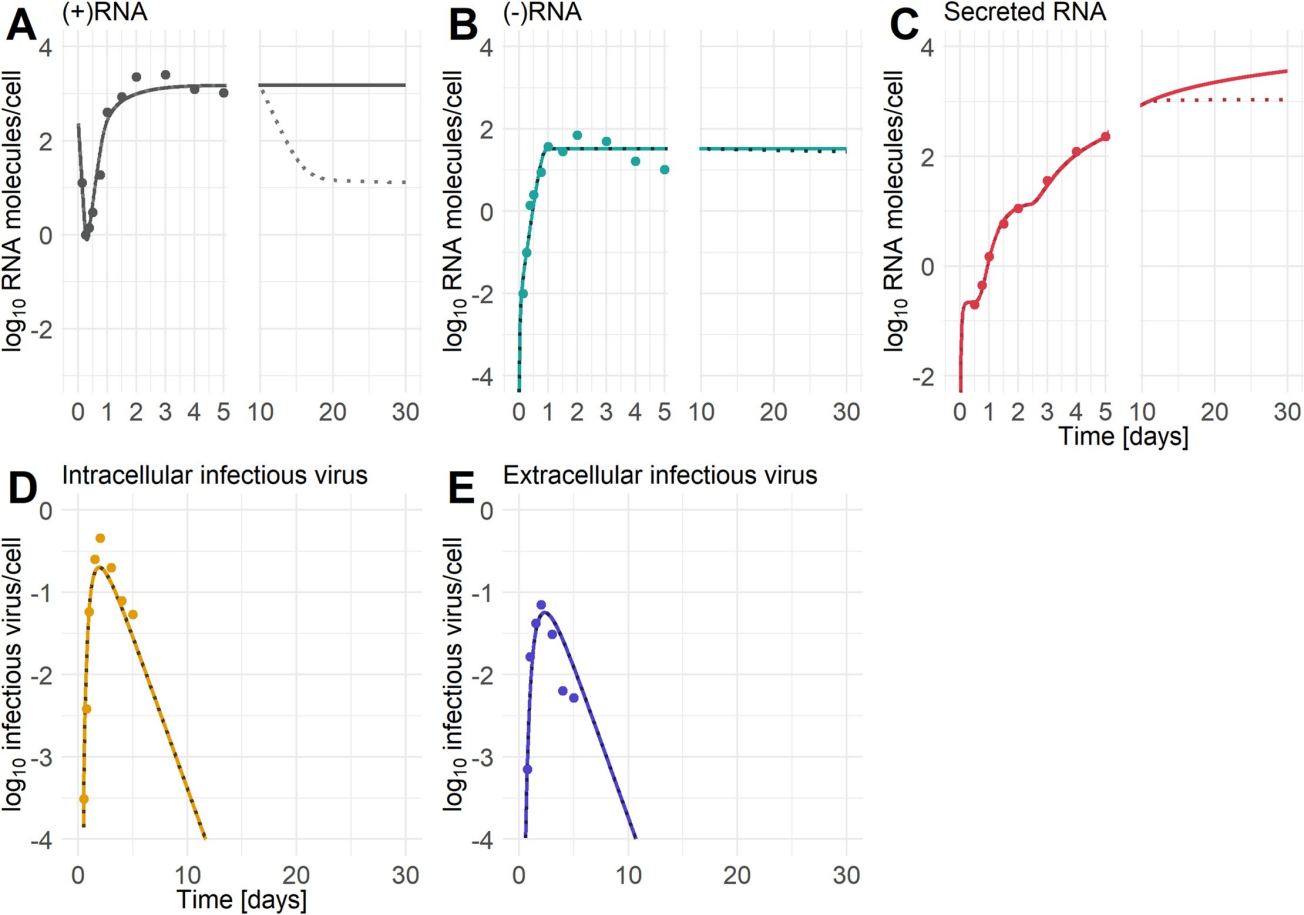
Effect of antiviral drug treatment given 10 dpi in the combined model (CM_4_). Here we assume the drug is the NS5A inhibitor daclatasvir and that it acts by inhibiting HCV RNA synthesis with an effectiveness *ε*_*R*_ = 0.99 and virus assembly with an effectiveness *ε*_*A*_ = 0.998. The solid line denotes the model’s prediction with no drug present, whereas the dotted line shows the model’s prediction after drug treatment is started on day 10 pi (see S1 Data).

Having developed a combined model of the viral lifecycle including virion assembly and secretion, we explored its use in predicting the effects of antiviral drug treatment. To this end, we simulated the effect of giving the direct acting antiviral daclatasvir 10 dpi when plus-strand and minus-strand RNA are in steady state. Daclatasvir interacts with the HCV NS5A protein and effectively blocks both viral replication (*ε*_*R*_ = 0.99) and assembly (*ε*_*A*_ = 0.998) and a single dose can lead to a 3 log decline in viral load *in vivo* [42,44,45]. To mimic daclatasvir’s *in vivo* activity, we multiplied the RNA synthesis rates, *r* and *α*, by the factor (1−*ε*_*R*_) and the virus assembly rate, *f*, by the factor (1−*ε*_*A*_), where the drug efficacies *ε*_*R*_ = 0.99 and *ε*_*A*_ = 0.998 were chosen based on previous estimates [42]. We found that plus-strand RNA decreased following drug administration (Fig 5A). However, due to the assumed lack of degradation of RNA species within the RC, the model exhibited no noticeable decrease of (-)RNA after drug administration Fig 5B. Intracellular and extracellular infectious virus were unaffected by the drug administration due to the late drug administration (Fig 5D and 5E). Lastly, under drug therapy secreted HCV RNA reached a constant level (Fig 5C) as the model did not include a mechanism for degradation of secreted exosomes.

## Discussion

Modeling HCV dynamics has a rich history and has been used for studying viral pathogenesis and spread, as well as the effects of antiviral treatment [20,44,46–50]. Some models have included intracellular events that occur during HCV replication and viral spread [20,30,31,51–53], but there is still a lack of detailed knowledge about these processes. The secretion of HCV RNA in the form of exosomes is currently not well understood, but experimental studies have shown that HCV RNA carrying exosomes derived from infected cells are able to infect naïve cells [15] as well as stimulate type I interferon responses from plasmacytoid dendritic cells [54]. Quintela et al. [30] introduced a mathematical model of intracellular HCV RNA replication that takes HCV RNA secretion into account. However, all plus-strand RNA species within their model can potentially be secreted and the model did not distinguish between secretion in exosomes from that in viral particles [30]. In the present study, we distinguish these two routes of secretion.

Experimentally, studying routes of secretion is challenging. Therefore, we introduced models that allowed us to compare three different hypotheses about the HCV RNA secretion routes: HCV RNA secretion from (i) the site of translation, (ii) the replication compartment, RC, and (iii) a combination of both. We also assumed that there may be a delay between viral infection and secretion of HCV RNA and examined different time delay models. By fitting these models to experimental data, we found that the preferred model included both sites of secretion with individual secretion route-specific model parameters.

In the experiments we analyzed, multiples copies of positive-strand HCV RNA entered the cell and on average about 13 copies were detected 3 hpi. Thus, it is not surprising that our secretion model suggested that HCV RNA was initially secreted from the cytoplasm, i.e., the site of translation. As HCV RNA is introduced into the cytoplasm following viral entry its secretion in exosomes as well as its degradation in the cytoplasm might be host cell defenses against viral infection. The loss of HCV RNA introduced by natural infection could be studied using a stochastic model as has been done for HIV [55]. However, in our model which is deterministic and is calibrated against an experimental system that involves the entry of multiple copies of HCV RNA extinction is not possible. Rather, from day 3 post-infection on positive-strand HCV RNA, which by that time has replicated, is mainly secreted from the RC. Before that plus-strand RNA within the RC is transported back to the cytoplasm at a per capita rate *θ* that is more than 10 times higher than its rate of secretion from the RC (see Table 4, SM parameters), suggesting early after infection this RNA is mainly used for protein synthesis in the cytoplasm.

Since experimental studies have shown that HCV-derived exosomes contain both plus-strand RNA as well as minus-strand RNA and possibly to a higher extent double stranded RNA [40], we studied the possibility that both plus- and minus-strand RNA are secreted from the cell. However, consistent with the experimental finding that the plus-strand to minus-strand HCV RNA ratio is 10:1 [56], we found that the minus-strand RNA contribution to the total secreted HCV RNA was negligible (S1 Text).

We also studied a model with HCV RNA secretion, presumably through exosomes, as well as through virus assembly and release. Fitting this model to data that included both secreted RNA and infectious virus release, we found that a function in which (after a delay) the rate of virion assembly decreased with time after infection best fit the *in vitro* data, suggesting that host or viral factors may limit the rate at which new infectious virions can be assembled. Further, the initial delay which we estimated to be about 6 hours long, would account for the early event in the viral lifecycle including production of the viral proteins needed for virion assembly.

The processes of HCV assembly, maturation, and release are closely linked to host cell lipid droplet assembly and the very-low-density lipoprotein (VLDL) pathway, as the HCV core protein is accumulating on cLD [4,57]. However, the precise structure of the HCV virion is still unknown due to its high lipid composition and the “lack of discernable surface features” [4]. Several if not all viral proteins are involved in virion assembly which is a tightly connected process between translation/replication and virus assembly/release [57]. It has been suggested that not only HCV proteins are involved in HCV assembly, maturation, and release but that several host factors also participate in HCV particle production, e.g. apolipoprotein (apo) E [57]. ApoE has been found to be a major component of cell culture grown HCV and primary-derived HCV [58]. Keum et al. [35] found that the apoE concentration associated with HCV particles decreases throughout infection, which might lead to a change in HCV infectivity. HCV particles produced early in infection are associated with a higher amount of apoE, while later in infection the HCV associated apoE concentration decreases. Catanese et al. [58] have shown that more copies of apoE were incorporated into the virion particle than the HCV structural protein E2 suggesting that apoE plays an important role in HCV attachment [58]. Deng et al. [59] suggest that the HCV glycoprotein E2 together with syntenin might be components of exosomes derived from HCV infected cells. Syntenin, a host factor regulating the exosome biogenesis, has been found to promote HCV E2 secretion, while the production of infectious virions remained unaffected and thus appears to be a parallel process to HCV exosome release. Deng et al. further suggest that HCV exosomes might support HCV escape from neutralizing E2-specific antibodies in chronic-phase patient sera and therefore might promote HCV infection [59]. Some host factors, such as the Y-box-binding protein (YB-1), have been found to restrict the process of viral assembly and release [7]. YB-1 has been shown to be a dynamic interacting partner of HCV NS3/4A, which impairs HCV replication, but promotes HCV particle production upon knockdown [60]. Furthermore, YB-1 has been suggested as regulating an equilibrium between HCV translation/replication and virus particle production [60]. However, little is known about its regulation in the HCV lifecycle and targeting a particular host factor that restricts viral assembly/release on the one hand but promotes viral replication on the other hand might lead to an increased secretion of HCV RNA with the ability to spread the infection. However, other host factors, such as miR-122, have been found balancing HCV RNA translation and replication. miR-122 increases HCV RNA levels available for synthesis in two different ways: (i) miR-122 stabilizes HCV RNA by preventing its degradation and (ii) it reduces the HCV RNA levels available for translation by dissolving the ribosome HCV RNA complex [61].

Another possible explanation for our finding of a decrease in infectious virus assembly and release with time might be the death of cells, which would also lead to less average release per unit time. However, such increased death was not seen in Keum et al. [35] until the last measurement time point at 5 dpi where increased cytotoxicity was detected in the infected cell cultures (Keum et al. Fig 2E). In the viral dynamics literature, there is a lack of models that take the complex processes of virion assembly and release into account. One exception is a model of influenza A virus developed by Heldt et al. [62] and these authors did not find a limitation in viral proteins or any viral components that are necessary for virus packaging and suggested that transport and budding processes might limit virus release [62]. However, unlike influenza A, HCV does not bud off the plasma membrane and instead is assembled intracellularly and then secreted.

Although not discussed in the main text, we also examined the possibility that cytosolic vRNA located at the site of translation might also serve as a source of vRNA that is packaged into virions. However, our model fits and AIC calculations showed that models where this possibility was absent were preferred. Instead, we found that models in which HCV RNA in the RC (or in close proximity to it) is used for virus assembly gave good fits to the data and were preferred based on AIC, consistent with the idea that cLDs associated with the endoplasmic reticulum membrane create an environment for HCV assembly. Overall, our analysis and observational data suggest that HCV replication and assembly are tightly linked processes within the membranous web [6,63]. Recently, by using correlative light and electron microscopy, Lee et al. [41] found that cLDs are wrapped by those endoplasmic reticulum membranes, which generate the RCs suggesting a close proximity and a short distance transport of vRNA from the site of HCV replication to assembly [41]. Due to the endoplasmic reticulum wrapped cLDs, which serve as sites of HCV assembly, the intracellular virus might be protected from decay, consistent with its degradation rate estimated by our model as being rather negligible.

It is becoming more and more evident that viruses use different transmission routes: the classic cell-free (receptor-mediated cell entry) and cell-to-cell (exosomal) transmission to infect neighboring cells [64,65]. Viral entry is a complex multi-step and highly regulated process involving viral proteins (HCV glycoproteins E1 and E2) and cellular (host) factors (e.g. CD81, claudin-1, occludin, SR-B1). *In vitro*, the viral entry process critically depends on the E1/E2-CD81 interaction as it has been shown that blocking CD81 or E1/E2 inhibits HCV infection [66–70]. In contrast, other studies [71–73], report that the presence of anti-E2 and anti-CD81 neutralizing antibodies do not block *in vitro* viral transmission completely and HCV is still transmitted to naïve target cells. This suggests not only multiple transmission routes but also the insufficiency in blocking viral entry as a therapeutic strategy [71–73]. It has been shown that anti-E2 treatment blocks HCV particle transmission but not exosomal HCV RNA transfer [15]. On the one hand, it is thought that receptor-mediated cell entry is crucial for infection initiation, since it has been shown that the presence of anti-envelope antibodies led to HCV resistance in HCV exposed patients [15]. On the other hand, exosomal cell-to-cell transfer seems to be an important feature of hepatitis C persistence and immune evasion and results in the resistance to neutralizing antibodies and complement attack [17,71,73–75]. Exosomes isolated from HCV-infected patients have been found to be enriched with CD81 and thus soluble CD81 may be of exosomal origin and represent an exosomal marker [14,76]. Increased soluble CD81 levels were significantly higher in patients with chronic hepatitis C [75].

Interestingly, our best-fit model (CM_4_) suggests secretion of HCV RNA from the site of translation starts almost immediately after infection, while Keum et al. [35] reported extracellular viral RNA was first detected 12 hpi. However, forcing a longer time delay, e.g. HCV RNA secretion starting at 8 hpi, increased the AIC from 198 (Table 6) to 240. An even larger increase in the AIC (281) was observed by starting HCV RNA secretion 11 hpi. One possible explanation for this discrepancy is that the amount of secreted HCV RNA per cell before 12 hpi is below the limit of detection as the amount measured at 12 hpi is only one molecule per 5 cells. Another possibility is that at these early time points with so few molecules being secreted, the secretion process is better described by a stochastic model than with our ODE model, but developing such models is outside the scope of this paper.

Taking our findings of the global sensitivity analysis into account, we found that HCV RNA synthesis and viral assembly might represent potent processes to target early and late in infection. Those processes are already targeted by direct acting antivirals such as sofosbuvir and mericitabine that inhibit the RNA-dependent RNA polymerase (HCV NS5B) and thus viral replication, as well as daclatasvir, ledipasvir, and elbasvir that inhibit the HCV NS5A protein, a phosphoprotein involved in both HCV replication and assembly [20,44]. A sustained virologic response above 95% can be achieved by blocking both, HCV replication with sofosbuvir and virus assembly and release with ledipasvir which represented the most sensitive processes in our global sensitivity analysis [77]. The NS5A inhibitor daclatasvir might lead to an almost complete shutoff of the HCV replication and spread due to its suggested ability to inhibit the formation of RCs and its ability to prevent HCV genome transfer to sites of virus assembly [78]. However, by using our model to study the effects of inhibiting both RNA synthesis and virus assembly, we found that blocking both processes led to a decrease in intracellular and extracellular virus concentration, as has been observed *in vitro* [42]. Nevertheless, as current direct acting antivirals are not 100% effective, our model predicted that HCV RNA was still being made and secreted at a low level. Our model was not designed for the *in vivo* situation and thus did not account for degradation or clearance of HCV RNA containing exosomes after their secretion from an infected cell. Interestingly, clinical trials aiming to predict the sustained virologic response rate to combination direct acting antiviral therapy regimens in HCV-infected patients have shown that some patients still had low level HCV RNA detectable at the end of therapy despite achieving a sustained virologic response [79,80]. Some possible explanations are that the detected HCV RNA might be non-infectious or very unfit due to mutations or defective due to aberrant virus assembly or because the HCV RNA was packaged in exosomes that poorly transmitted infection, i.e. had an *R*_0_<1 [79].

In summary, in the present study we compared several mathematical models of intracellular HCV replication coupled to HCV RNA secretion and/or virus assembly and release. Using a model that did not distinguish between HCV RNA secretion in exosomes or viral particles, we found that initially HCV RNA from the cytoplasm served as the main source of secreted RNA, but after a delay the HCV RNA from the site of translation and the RC served equally as the sources for secreted HCV RNA. By expanding our model to explicitly include HCV assembly and release, we found that a model in which after a delay the rate of viral assembly/release decreased with the additional amount of time a cell had been infected fit the data best, suggesting that these processes might be limited by the availability of viral components and/or host factors involved in assembly and release. Alternatively, there might be host restriction factors that are induced following infection that limit virus particle production. Moreover, if the rate of HCV RNA replication and the amount of intracellular HCV RNA increase with time after infection, this might increase the secretion of HCV RNA in exosomes, which can evade host antibody responses to viral envelope proteins, such as E2, and have the ability to infect naïve cells and thus spread the infection. HCV dynamics models have focused on HCV RNA dispersion in viral particles. Our work suggests that HCV RNA spread in exosomes is also likely and further research concerning exosomal secretion, its infective potential, as well as its contribution to viral spread is needed.

## Supporting information

S1 Fig HCV secretion model fits. Best fits of the HCV RNA secretion models for the three different time delay functions: ramp-up secretion (type 1 models), simple time delayed secretion (type 2 models), exponential decreasing HCV RNA secretion (type 3 models). A) Best-fit model for secretion exclusively from the site of translation. B) Best-fit model for secretion exclusively from the RC. C) Best-fit model for equal (*τ*_*T*_ = *τ*_*R*_ and *ρ*_*T*_ = *ρ*_*R*_) secretion from both sites, the site of translation and the RC. D) Best-fit model for individual (*τ*_*T*_≠*τ*_*R*_ and *ρ*_*T*_≠*ρ*_*R*_) secretion from both sites, the site of translation and the RC. [(-)RNA = minus-stranded RNA, (+)RNA = plus-stranded RNA, sRNA = secreted HCV RNA (see S1 Data)]. Data has been taken from [35] Fig 1A–1C. See S1 and S2 Tables for parameter information.
(TIFF)

S2 Fig Best fits of the HCV RNA secretion models. A) Best-fit model for secretion exclusively from the site of translation. B) Best-fit model for secretion exclusively from the RC. C) Best-fit model for equal (*τ*_*T*_ = *τ*_*R*_ and *ρ*_*T*_ = *ρ*_*R*_) secretion from both sites, the site of translation and the RC (left), as well as sources of secreted HCV RNA (right). D) Best-fit model for individual (*τ*_*T*_≠*τ*_*R*_ and *ρ*_*T*_≠*ρ*_*R*_) secretion from both sites, the site of translation and the RC (left), as well as sources of secreted HCV RNA (right). [(-)RNA = minus-stranded RNA, (+)RNA = plus-stranded RNA, sRNA = secreted HCV RNA (see S1 Data)]. Data has been taken from [35] Fig 1A–1C. See S1 Table for parameter information.
(TIFF)

S3 Fig Parameter identifiability profile for the best-fit HCV secretion model. Parameter identifiability profile for the best-fit HCV secretion model (SM_T1≠R1_) that considers independent HCV RNA secretion (*τ*_*T*_≠*τ*_*R*_, *ρ*_*T*_≠*ρ*_*R*_, *k*_*T*_≠*k*_*R*_). The x-axis shows the scanned parameter profile (as log_10_ values), y-axis shows the corresponding log-likelihood values [ΔL(P) is the difference of the log likelihood value], the red dot shows the estimated parameter value and the red line describes the statistical 95% threshold (95% confidence intervals are listed in Table 4, see S1 Data for details). A parameter is identifiable if the black parameter profile line is crossing the statistical threshold (the 95% confidence interval is finite).
(TIFF)

S4 Fig Parameter identifiability profile for the best-fit HCV assembly model (AM_4_). The x-axis shows the scanned parameter profile (as log_10_ values), y-axis shows the corresponding log-likelihood values [ΔL(P) is the difference of the log likelihood value], the red dot shows the estimated parameter value and the red line describes the statistical 95% threshold (95% confidence intervals are listed in Table 4, see S1 Data for details). A parameter is identifiable if the black parameter profile line is crossing the statistical threshold (the 95% confidence interval is finite).
(TIFF)

S5 Fig Parameter identifiability profile for the best-fit HCV RNA secretion and virus assembly model (CM_4_). The x-axis shows the scanned parameter profile (as log_10_ values), y-axis shows the corresponding log-likelihood values [ΔL(P) is the difference of the log likelihood value], the red dot shows the estimated parameter value and the red line describes the statistical 95% threshold (95% confidence intervals are listed in Table 4, see S1 Data for details). A parameter is identifiable if the black parameter profile line is crossing the statistical threshold (the 95% confidence interval is finite).
(TIFF)

S6 Fig (+) and (-)RNA secretion model. Schematic illustration of the intracellular HCV RNA replication extended by *ρ*_*C*_, where (-)RNA from the RC serves as a source of HCV RNA secretion. For more details, see Fig 1.
(TIF)

S7 Fig AICs of the (+) and (-)RNA secretion models. Best-fit model AICs of the HCV (+) and (-)RNA secretion models when the parameter *k*_*ρ*_ determining the rate of ramp-up or limitation was varied. [▲ = ramp-up models, ■ = simple delay models, ● = exponential decrease models; blue = (-)RNA secretion from the RC, green = equal (+)RNA secretion from site of translation and (-)RNA secretion from the RC (*τ*_*T*_ = *τ*_*C*_, *ρ*_*T*_ = *ρ*_*C*_), yellow = individual (+)RNA secretion from site of translation and (-)RNA secretion from the RC (*τ*_*T*_≠*τ*_*C*_, *ρ*_*T*_≠*ρ*_*C*_), red = equal (+)RNA secretion from site of translation and (+) and (-) RNA secretion from the RC (*τ*_*T*_ = *τ*_*R*_ = *τ*_*C*_, *ρ*_*T*_ = *ρ*_*R*_ = *ρ*_*C*_), black = individual (+)RNA secretion from site of translation and (+) and (-)RNA secretion from the RC (*τ*_*T*_≠*τ*_*R*_≠*τ*_*C*_, *ρ*_*T*_≠*ρ*_*R*_≠*ρ*_*C*_) (see S1 Data)]. For the best model for each HCV RNA secretion route and corresponding time delay function see S3 Table.
(TIFF)

S8 Fig (+) and (-)RNA secretion model fit. A) Best-fit model for individual HCV (+)RNA and (-)RNA secretion from site of translation and the RC (*τ*_*T*_≠*τ*_*R*_≠*τ*_*C*_, *ρ*_*T*_≠*ρ*_*R*_≠*ρ*_*C*_, and *k*_*T*_≠*k*_*R*_≠*k*_*C*_). B) Sources of secreted HCV RNA. C) Ratios of intracellular HCV RNA species. D) Time delay functions. [(-)RNA = minus-stranded RNA, (+)RNA = plus-stranded RNA, sRNA = secreted HCV RNA (see S1 Data)]. Data has been taken from [35] Fig 1A–1C. See S4 Table for parameter information.
(TIFF)

S9 Fig Parameter identifiability profile for the best-fit (+) and (-)HCV secretion model. Parameter identifiability profile for the best-fit (+) and (-)HCV secretion model (SM_T1≠R1≠M1_) that considers independent HCV RNA secretion (*τ*_*T*_≠*τ*_*R*_≠*τ*_*C*_, *ρ*_*T*_≠*ρ*_*R*_≠*ρ*_*C*_, *k*_*T*_≠*k*_*R*_≠*k*_*C*_). The x-axis shows the scanned parameter profile (as log_10_ values), y-axis shows the corresponding log-likelihood values [ΔL(P) is the difference of the log likelihood value], the red dot shows the estimated parameter value and the red line describes the statistical 95% threshold (95% confidence intervals are listed in S4 Table, see S1 Data for details). A parameter is identifiable if the black parameter profile line is crossing the statistical threshold (the 95% confidence interval is finite).
(TIFF)

S10 Fig Global sensitivity of the CM_4_ model parameters. Global sensitivity analysis performed for model CM_4_ showing the total-order sensitivities for all model parameters for two different time points: 1 (A, C, E) and 3 (B, D, F, G, H) days post infection (dpi). The red line represents a threshold, a so-called negative control or dummy parameter that does not appear in the mathematical model equations, where sensitivities above the line are considered as relevant while those below are negligible (see Methods section and S1 Data for details). Significant differences of the total sensitivity of a model parameter to the threshold have been calculated by performing a t-Test (p-values: *** ≤0.001,**≤0.01,*≤0.05) (see S2 Text for more information).
(TIFF)

S1 Table Parameter values of secretion models with one HCV (+)RNA secretion route. Parameter values of the best-fit models with a delayed ramp-up secretion (type 1 models: SM_T1_ and SM_R1_), simple time delayed secretion (type 2 models: SM_T2_ and SM_R2_), and an exponential decreasing secretion (type 3 models: SM_T3_ and SM_R3_).
(DOCX)

S2 Table Parameter values of secretion models with both HCV (+)RNA secretion route. Parameter values of the best-fit models with a delayed ramp-up secretion (type 1 models: SM_T1 = R1_ and SM_T1≠R1_), simple time delayed secretion (type 2 models: SM_T2 = R2_ and SM_T2≠R2_), and an exponential decreasing secretion (type 3 models: SM_T3 = R3_ and SM_T3≠R3_). Note that for models where HCV RNA is secreted from both routes (the site of translation and the RC) we discriminate between two different cases: (i) secretion specific model parameters are the same for both secretion routes, i.e. *τ*_*T*_ = *τ*_*R*_ and *ρ*_*T*_ = *ρ*_*R*_ (SM_Ti = Ri_ with i = 1,2,3), or (ii) secretion specific model parameters are individual for both secretion routes, i.e. *τ*_*T*_≠*τ*_*R*_ and *ρ*_*T*_≠*ρ*_*R*_ (SM_Ti≠Ri_ with i = 1,2,3). Parameter values in [] show 95% confidence intervals, while values marked with * were kept fixed throughout the profile likelihood estimation.
(DOCX)

S3 TableModel comparison of the (+) and (-)RNA secretion models.AICs, number of estimated model parameters (#P), time delay parameters ($k_{\rho_{i}},\;\tau_{\rho_{i}},\;\rho_{i}$) values of the best-fit models that take into account the secretion of (-)RNA and were fitted to measurements of plus-strand RNA, minus-strand RNA, and secreted HCV RNA in Keum et al. [35]. The model with the lowest AIC is highlighted in beige and the lowest AIC for each model is shown in bold.(DOCX)

S4 Table Parameter values of the (+) and (-)RNA secretion model. Parameter values of the best-fit model that consider the secretion of HCV (+)RNA and (-)RNA (S2 and S4 Figs, S3 Table) with a delayed ramp-up secretion. Note that every secretion route is individual and hence, *k*_*T*_≠*k*_*R*_≠*k*_*C*_, *τ*_*T*_≠*τ*_*R*_≠*τ*_*C*_, and *ρ*_*T*_≠*ρ*_*R*_≠*ρ*_*C*_. Parameter values in [] show 95% confidence intervals, while values marked with * were kept fixed throughout the profile likelihood estimation. The degradation rate of HCV RNA within the RC was set to *μ*_*R*_ = 0 (see main text).
(DOCX)

S1 Text Minus-strand and plus-strand HCV RNA as secretion sources. (DOCX)

S2 Text Sensitivity analysis of the best fit model (CM_4_). (DOCX)

S1 Data (XLSX)
