# Supplementary figures and images for: Mathematical modeling of hepatitis C RNA replication, exosome secretion and virus release
Source: PLoS Comput Biol. 2020 Nov 5;16(11):e1008421. doi: 10.1371/journal.pcbi.1008421 (PMC7671504; doi:10.1371/journal.pcbi.1008421)

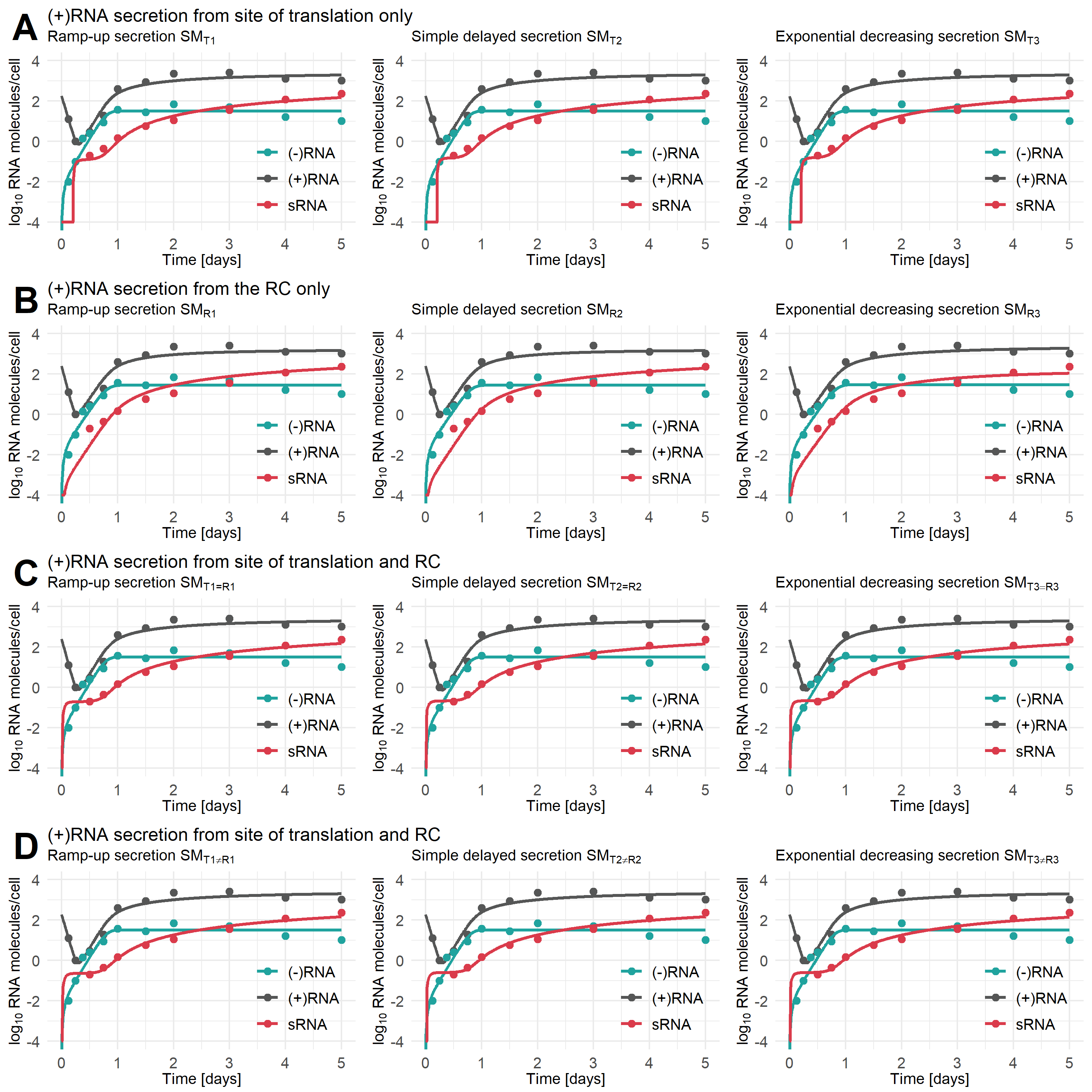

Supplement: S1 Fig — Best fits of the HCV RNA secretion models for the three different time delay functions: ramp-up secretion (type 1 models), simple time delayed secretion (type 2 models), exponential decreasing HCV RNA secretion (type 3 models). A) Best-fit model for secretion exclusively from the site of translation. B) Best-fit model for secretion exclusively from the RC. C) Best-fit model for equal (τT = τR and ρT = ρR) secretion from both sites, the site of translation and the RC. D) Best-fit model for individual (τT≠τR and ρT≠ρR) secretion from both sites, the site of translation and the RC. [(-)RNA = minus-stranded RNA, (+)RNA = plus-stranded RNA, sRNA = secreted HCV RNA (see S1 Data)]. Data has been taken from [35] Fig 1A–1C. See S1 and S2 Tables for parameter information. (TIFF) [file pcbi.1008421.s001.tiff]

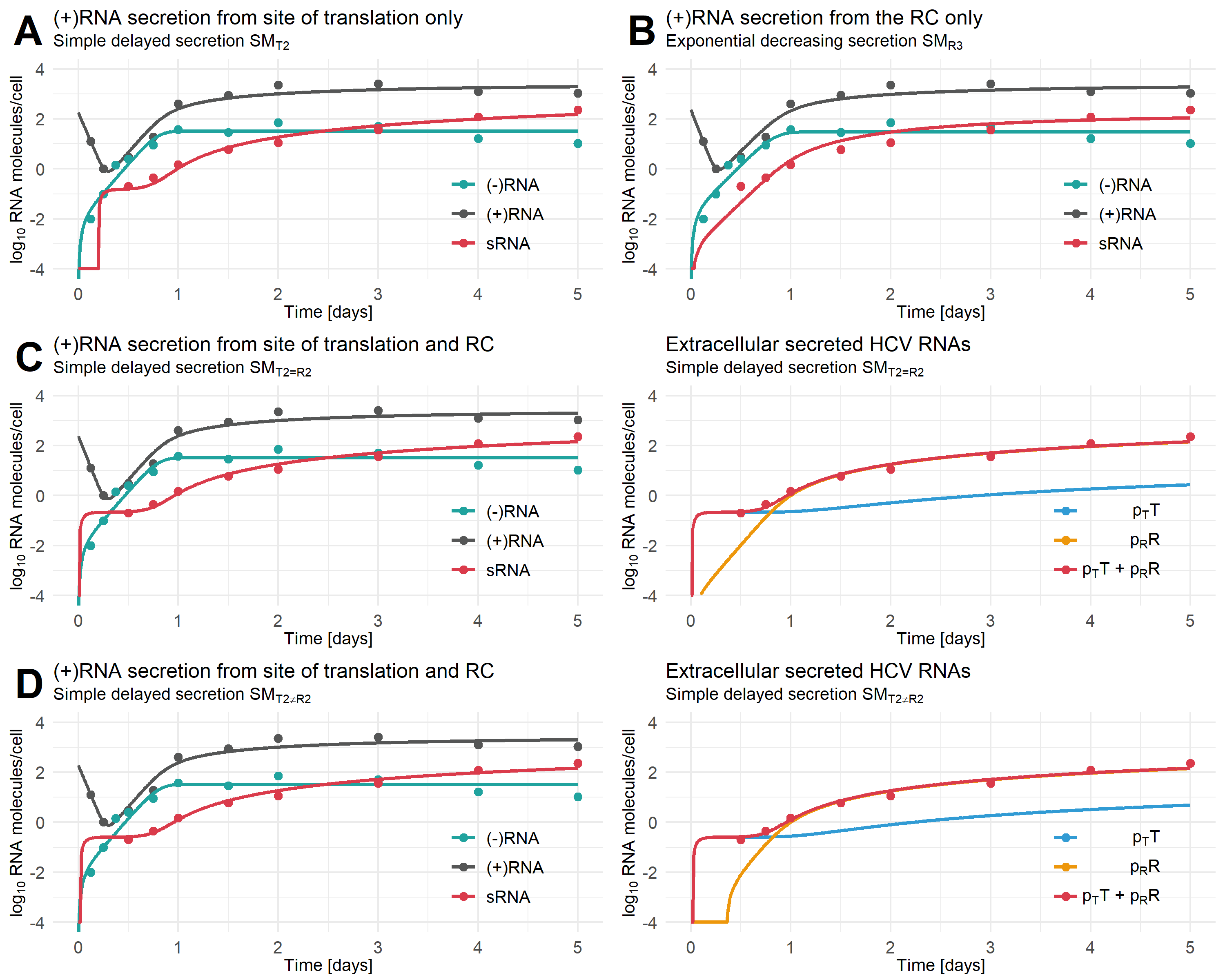

Supplement: S2 Fig — A) Best-fit model for secretion exclusively from the site of translation. B) Best-fit model for secretion exclusively from the RC. C) Best-fit model for equal (τT = τR and ρT = ρR) secretion from both sites, the site of translation and the RC (left), as well as sources of secreted HCV RNA (right). D) Best-fit model for individual (τT≠τR and ρT≠ρR) secretion from both sites, the site of translation and the RC (left), as well as sources of secreted HCV RNA (right). [(-)RNA = minus-stranded RNA, (+)RNA = plus-stranded RNA, sRNA = secreted HCV RNA (see S1 Data)]. Data has been taken from [35] Fig 1A–1C. See S1 Table for parameter information. (TIFF) [file pcbi.1008421.s002.tiff]

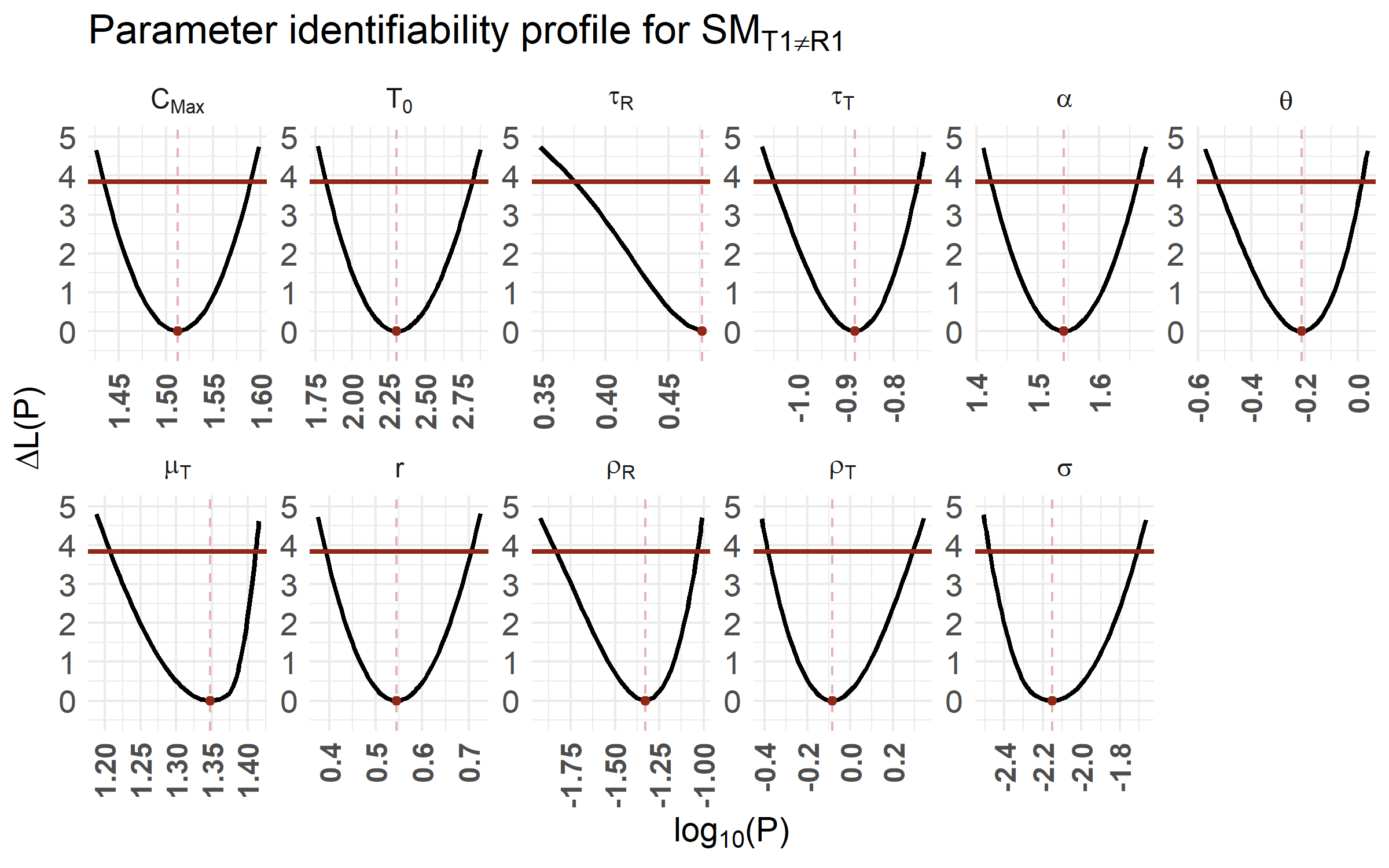

Supplement: S3 Fig — Parameter identifiability profile for the best-fit HCV secretion model (SMT1≠R1) that considers independent HCV RNA secretion (τT≠τR, ρT≠ρR, kT≠kR). The x-axis shows the scanned parameter profile (as log10 values), y-axis shows the corresponding log-likelihood values [ΔL(P) is the difference of the log likelihood value], the red dot shows the estimated parameter value and the red line describes the statistical 95% threshold (95% confidence intervals are listed in Table 4, see S1 Data for details). A parameter is identifiable if the black parameter profile line is crossing the statistical threshold (the 95% confidence interval is finite). (TIFF) [file pcbi.1008421.s003.tiff]

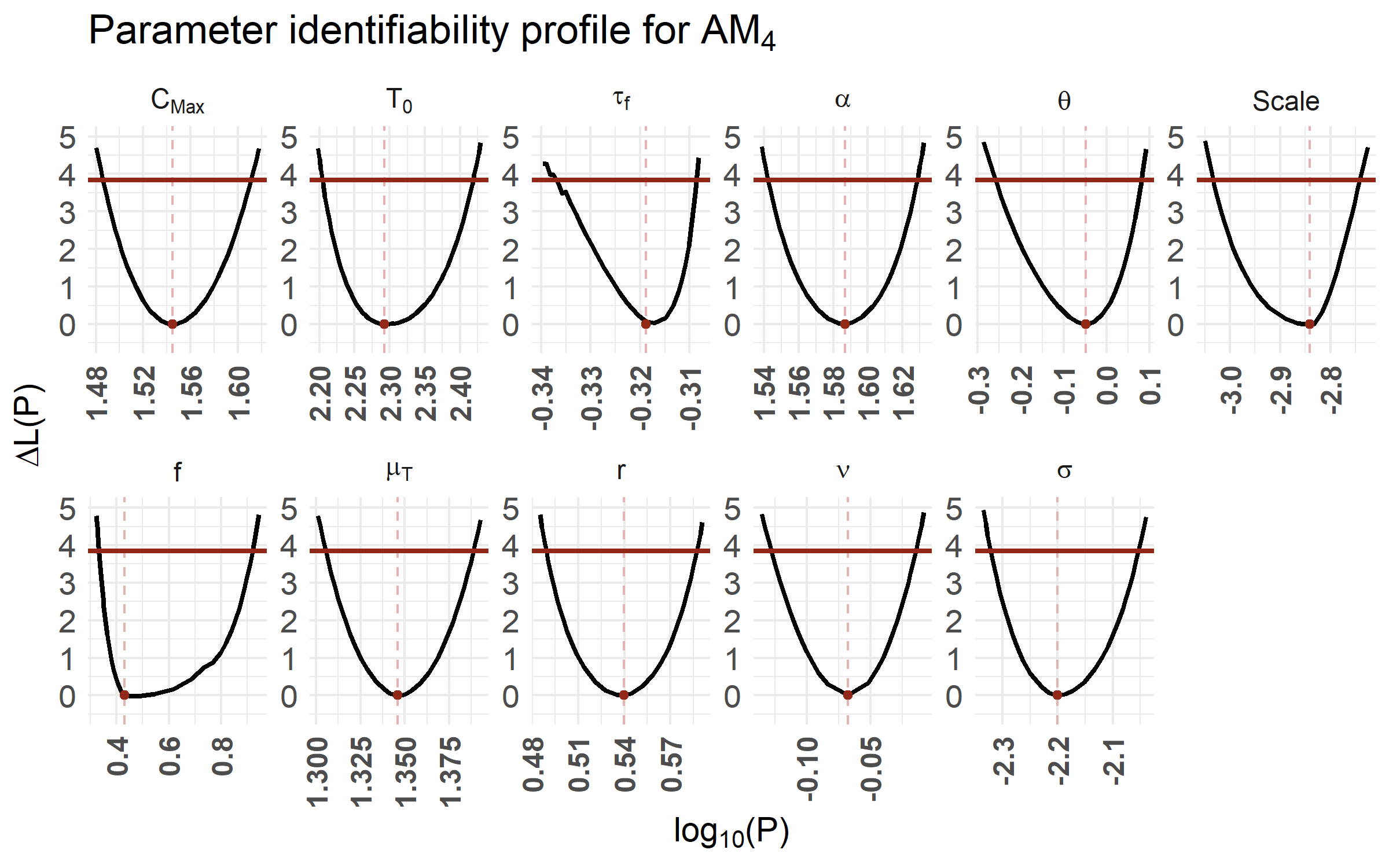

Supplement: S4 Fig — The x-axis shows the scanned parameter profile (as log10 values), y-axis shows the corresponding log-likelihood values [ΔL(P) is the difference of the log likelihood value], the red dot shows the estimated parameter value and the red line describes the statistical 95% threshold (95% confidence intervals are listed in Table 4, see S1 Data for details). A parameter is identifiable if the black parameter profile line is crossing the statistical threshold (the 95% confidence interval is finite). (TIFF) [file pcbi.1008421.s004.tiff]

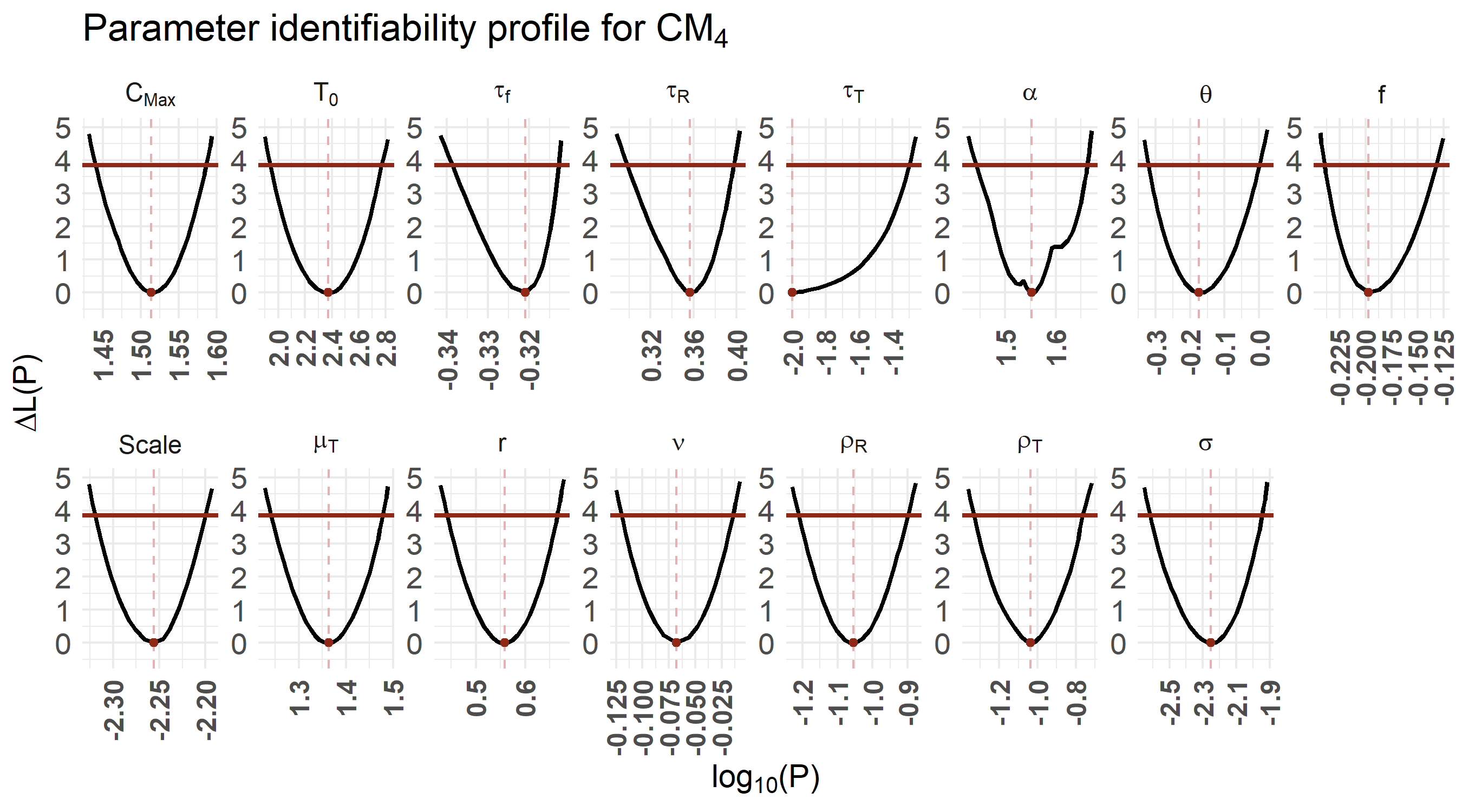

Supplement: S5 Fig — The x-axis shows the scanned parameter profile (as log10 values), y-axis shows the corresponding log-likelihood values [ΔL(P) is the difference of the log likelihood value], the red dot shows the estimated parameter value and the red line describes the statistical 95% threshold (95% confidence intervals are listed in Table 4, see S1 Data for details). A parameter is identifiable if the black parameter profile line is crossing the statistical threshold (the 95% confidence interval is finite). (TIFF) [file pcbi.1008421.s005.tiff]

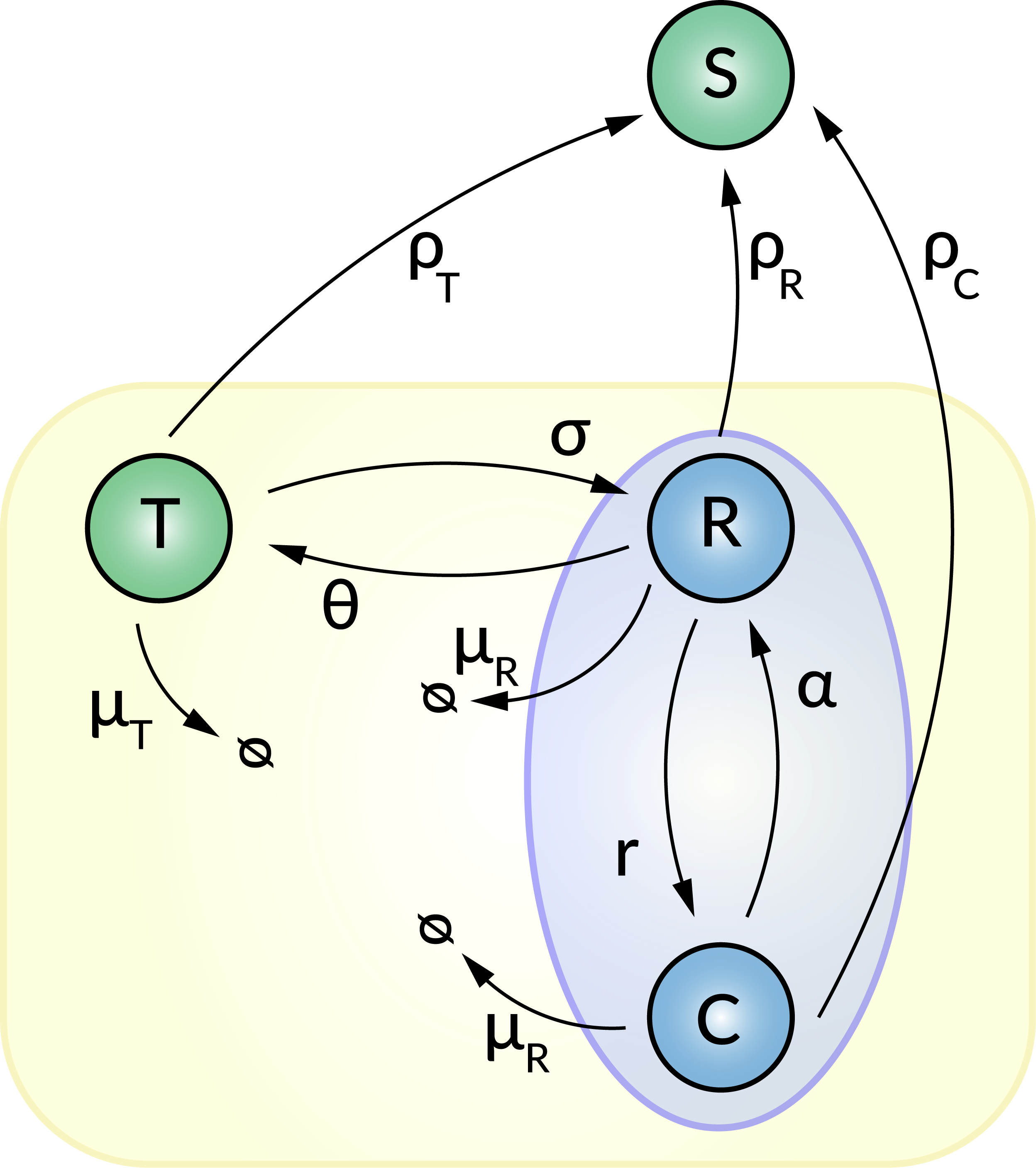

Supplement: S6 Fig — Schematic illustration of the intracellular HCV RNA replication extended by ρC, where (-)RNA from the RC serves as a source of HCV RNA secretion. For more details, see Fig 1. (TIF) [file pcbi.1008421.s006.tif]

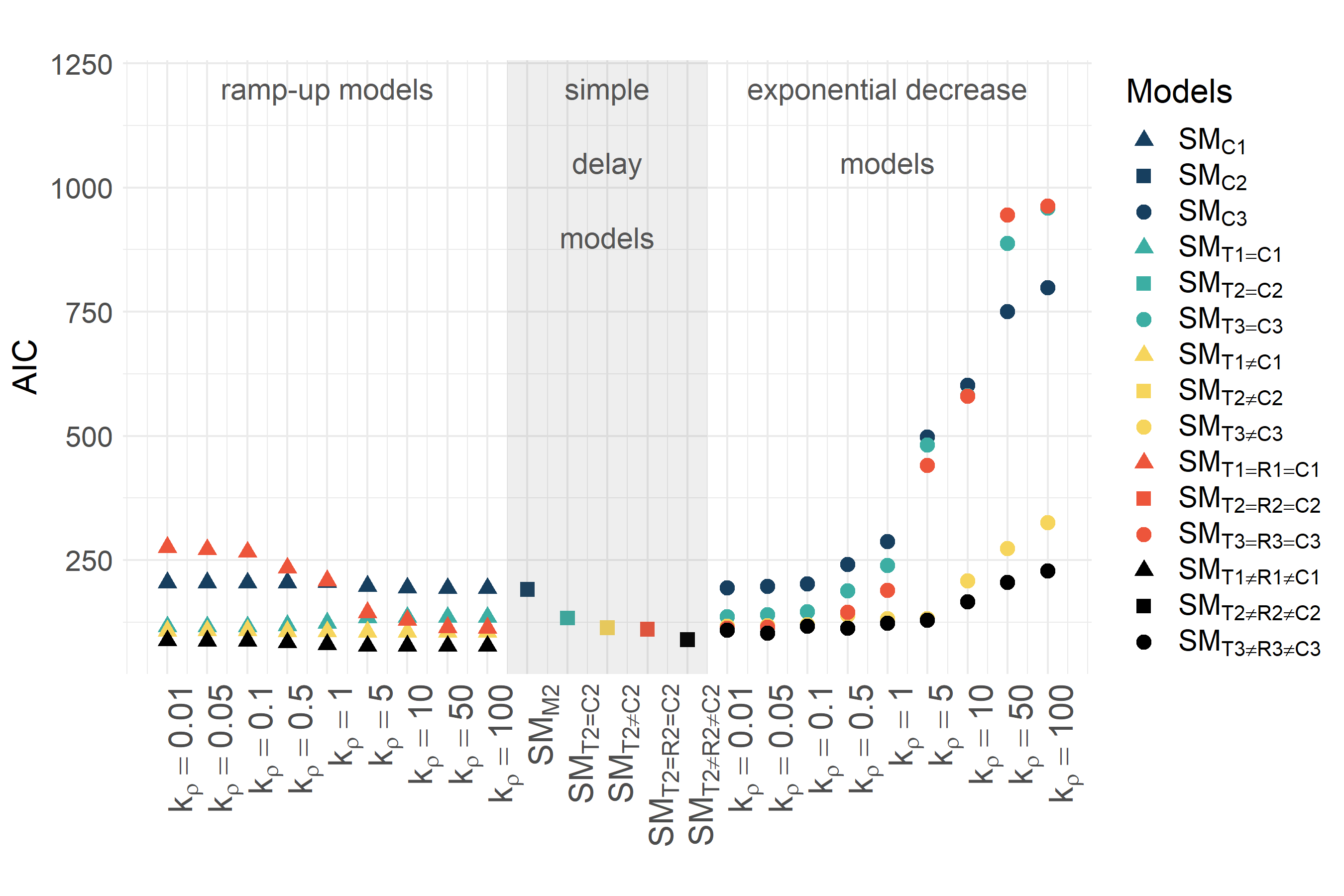

Supplement: S7 Fig — Best-fit model AICs of the HCV (+) and (-)RNA secretion models when the parameter kρ determining the rate of ramp-up or limitation was varied. [▲ = ramp-up models, ■ = simple delay models, ● = exponential decrease models; blue = (-)RNA secretion from the RC, green = equal (+)RNA secretion from site of translation and (-)RNA secretion from the RC (τT = τC, ρT = ρC), yellow = individual (+)RNA secretion from site of translation and (-)RNA secretion from the RC (τT≠τC, ρT≠ρC), red = equal (+)RNA secretion from site of translation and (+) and (-) RNA secretion from the RC (τT = τR = τC, ρT = ρR = ρC), black = individual (+)RNA secretion from site of translation and (+) and (-)RNA secretion from the RC (τT≠τR≠τC, ρT≠ρR≠ρC) (see S1 Data)]. For the best model for each HCV RNA secretion route and corresponding time delay function see S3 Table. (TIFF) [file pcbi.1008421.s007.tiff]

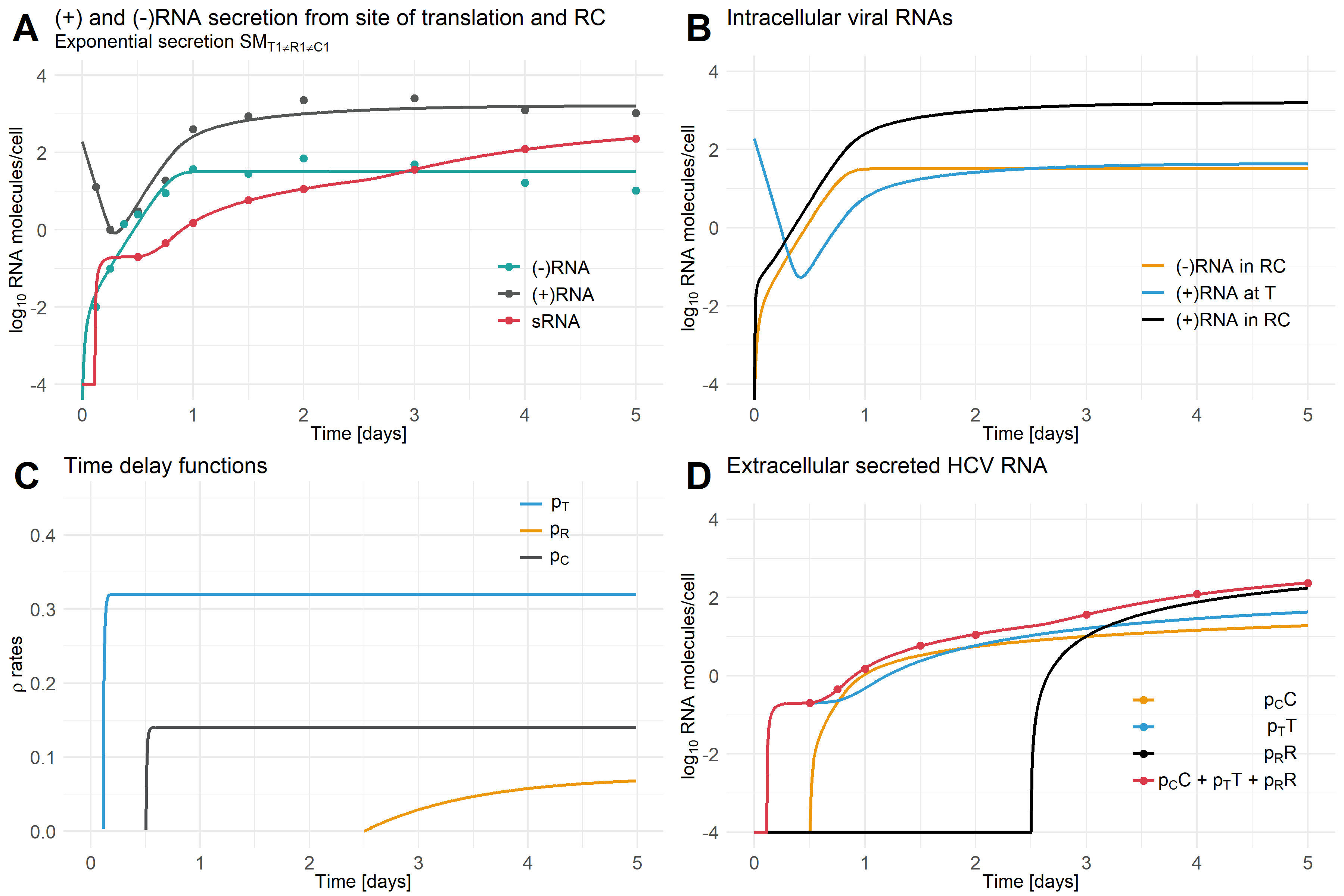

Supplement: S8 Fig — A) Best-fit model for individual HCV (+)RNA and (-)RNA secretion from site of translation and the RC (τT≠τR≠τC, ρT≠ρR≠ρC, and kT≠kR≠kC). B) Sources of secreted HCV RNA. C) Ratios of intracellular HCV RNA species. D) Time delay functions. [(-)RNA = minus-stranded RNA, (+)RNA = plus-stranded RNA, sRNA = secreted HCV RNA (see S1 Data)]. Data has been taken from [35] Fig 1A–1C. See S4 Table for parameter information. (TIFF) [file pcbi.1008421.s008.tiff]

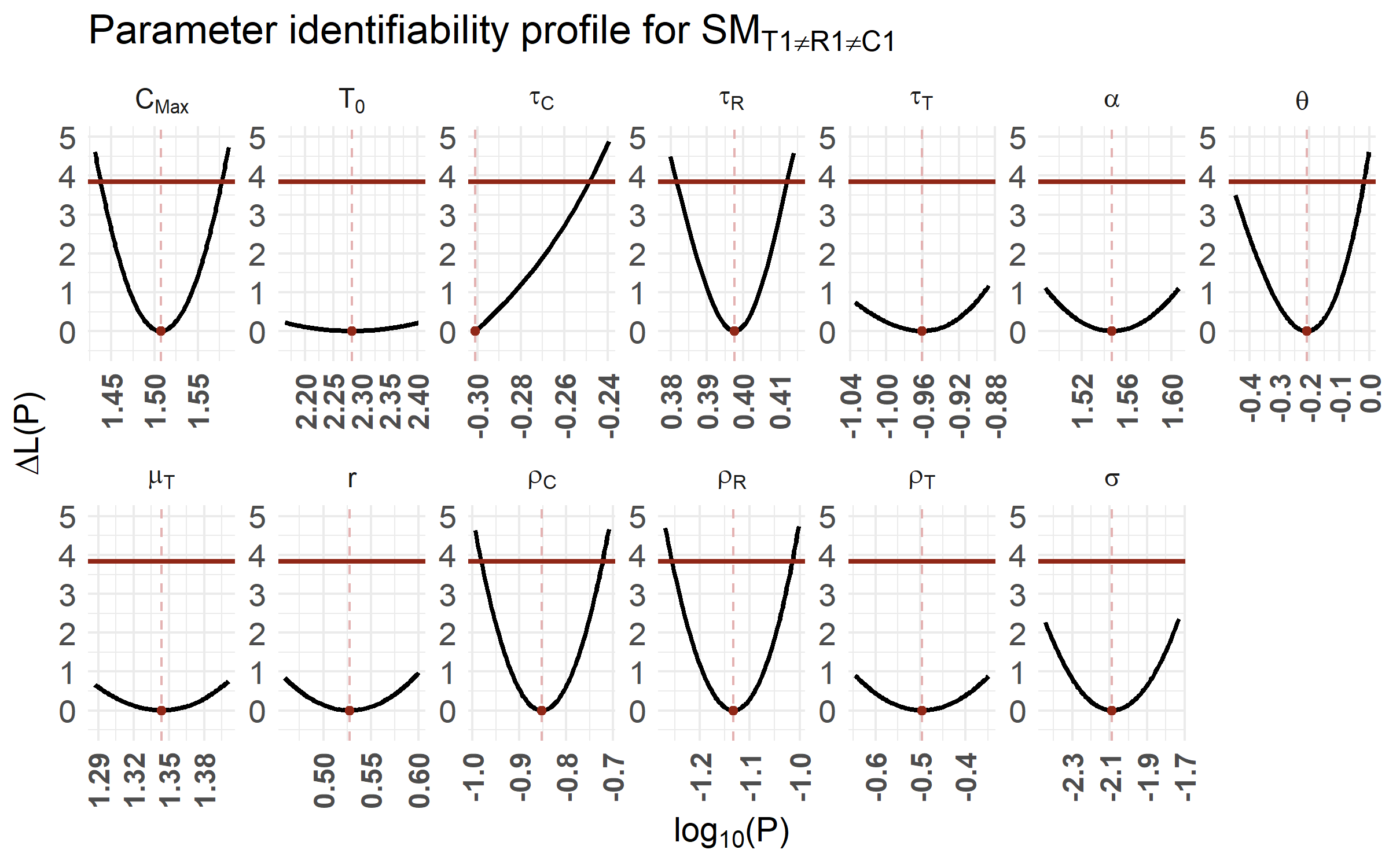

Supplement: S9 Fig — Parameter identifiability profile for the best-fit (+) and (-)HCV secretion model (SMT1≠R1≠M1) that considers independent HCV RNA secretion (τT≠τR≠τC, ρT≠ρR≠ρC, kT≠kR≠kC). The x-axis shows the scanned parameter profile (as log10 values), y-axis shows the corresponding log-likelihood values [ΔL(P) is the difference of the log likelihood value], the red dot shows the estimated parameter value and the red line describes the statistical 95% threshold (95% confidence intervals are listed in S4 Table, see S1 Data for details). A parameter is identifiable if the black parameter profile line is crossing the statistical threshold (the 95% confidence interval is finite). (TIFF) [file pcbi.1008421.s009.tiff]

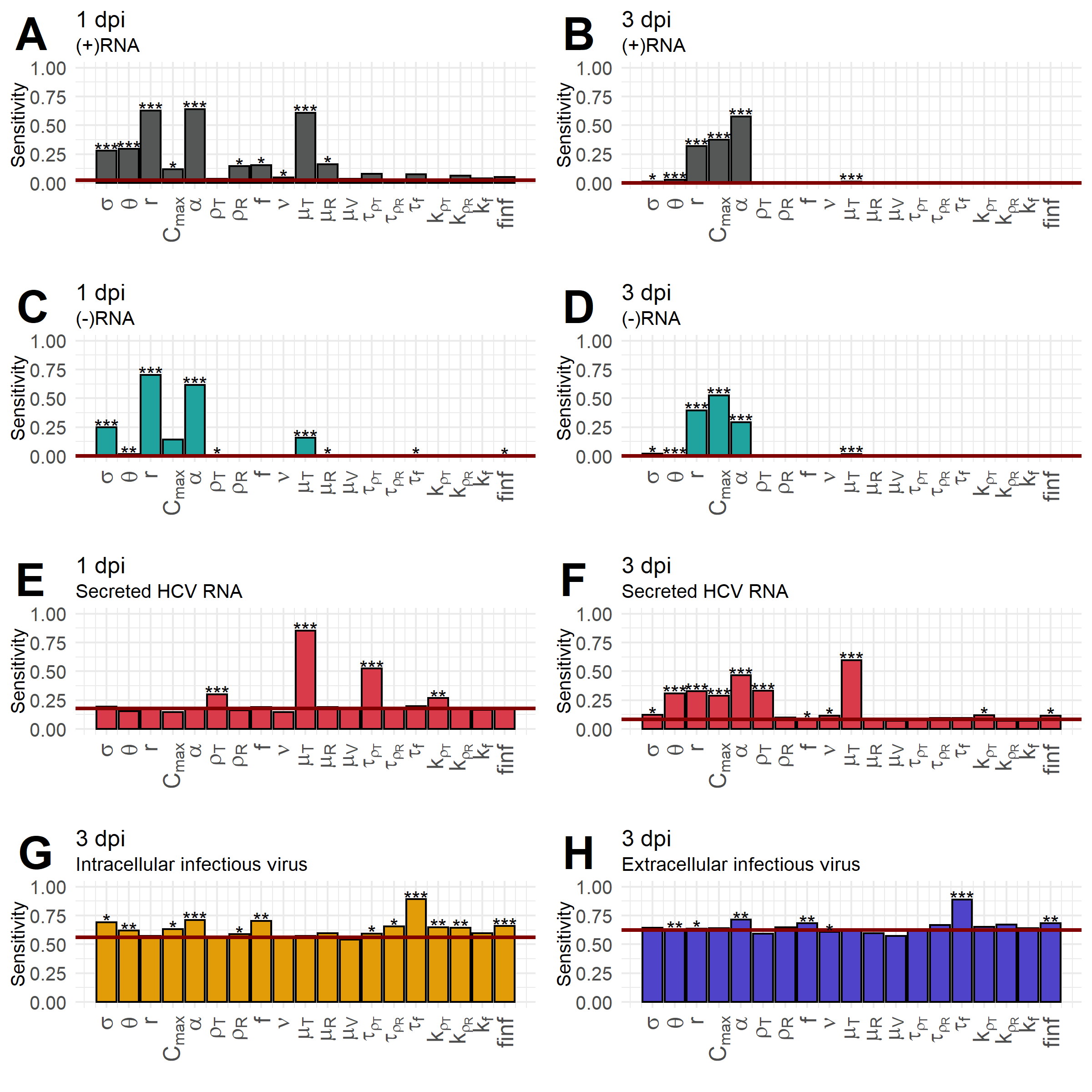

Supplement: S10 Fig — Global sensitivity analysis performed for model CM4 showing the total-order sensitivities for all model parameters for two different time points: 1 (A, C, E) and 3 (B, D, F, G, H) days post infection (dpi). The red line represents a threshold, a so-called negative control or dummy parameter that does not appear in the mathematical model equations, where sensitivities above the line are considered as relevant while those below are negligible (see Methods section and S1 Data for details). Significant differences of the total sensitivity of a model parameter to the threshold have been calculated by performing a t-Test (p-values: *** ≤0.001,**≤0.01,*≤0.05) (see S2 Text for more information). (TIFF) [file pcbi.1008421.s010.tiff]
