# Supplementary material for: Mathematical modeling of hepatitis C RNA replication, exosome secretion and virus release
Source: PLoS Comput Biol. 2020 Nov 5;16(11):e1008421. doi: 10.1371/journal.pcbi.1008421 (PMC7671504; doi:10.1371/journal.pcbi.1008421)
Supplement: S1 Table — Parameter values of the best-fit models with a delayed ramp-up secretion (type 1 models: SMT1 and SMR1), simple time delayed secretion (type 2 models: SMT2 and SMR2), and an exponential decreasing secretion (type 3 models: SMT3 and SMR3). (DOCX) [file pcbi.1008421.s011.docx]

| Parameter | Description | SM_T1_ | SM_T2_ | SM_T3_ | SM_R1_ | SM_R2_ | SM_R3_ | Unit |
| --- | --- | --- | --- | --- | --- | --- | --- | --- |
| $\boldsymbol{AIC}$ |  | 113.4 | **111.4** | 113.7 | 230.6 | 228.5 | **204.5** |  |
| $\boldsymbol{\rho}_{\boldsymbol{T}}$ | $S$ secretion rate | 1000 [0.001, 1000] | 324.5 [10.2, 1000] | 1.5 [1.0, 2.4] | - | - | - | $d^{-1}$ |
| $\boldsymbol{\rho}_{\boldsymbol{R}}$ |  | - | - | - | 0.045 [0.036, 0.056] | 0.045 [0.036, 0.056] | 0.1 [0.08, 0.13] | $d^{-1}$ |
| $\boldsymbol{\tau}_{\boldsymbol{\rho}_{\boldsymbol{T}}}$ | $S$ secretion delay | 0.3 [0.01, 0.36] | 0.3 [0.01, 0.36] | 0.18 [0.14, 0.5] | - | - | - | $d$ |
| $\boldsymbol{\tau}_{\boldsymbol{\rho}_{\boldsymbol{R}}}$ |  | - | - | - | 0.01 [0.01, 0.18] | 0.01 [0.01, 0.19] | 0.01 [0.01, 0.2] | $d$ |
| $\boldsymbol{k}_{\boldsymbol{\rho}_{\boldsymbol{T}}}\boldsymbol{=}\boldsymbol{k}_{\boldsymbol{\rho}_{\boldsymbol{R}}}$ | $S$ secretion rate parameter | 100 * | - | 0.01 * | 100 * | - | 0.5 * | $d^{-1}$ |
| $\boldsymbol{T}_{\boldsymbol{0}}$ | Initial number of RNAs transfected into the cell | 201 [150, 250] | 218 [150, 250] | 179 [60.2, 250] | 250 [72.6, 250] | 250 [72.8, 250] | 239 [150, 250] | molecules/cell |
| $\boldsymbol{C}_{\boldsymbol{max}}$ | Maximal number of $C$ | 32.3 [27.0, 38.6] | 32.3 [27.0, 38.6] | 32.3 [27.0, 38.4] | 27.8 [23.3, 33.2] | 27.8 [23.3, 33.2] | 29.7 [25.0, 35.5] | molecules/cell |
| $\boldsymbol{\sigma}$ | Rate of transfer of $T$ to the RC | 0.007 [0.002, 0.02] | 0.007 [0.002, 0.02] | 0.008 [0.003, 0.02] | 0.015 [0.008, 0.05] | 0.015 [0.008, 0.046] | 0.014 [0.005, 0.04] | $d^{-1}$ |
| $\boldsymbol{\theta}$ | Rate of transfer of $R$to the cytoplasm | 0.027 [0.02, 1000] | 0.028 [0.02, 0.08] | 0.4 [0.001, 0.8] | 0.69 [0.001, 1.16] | 0.69 [0.001, 1.2] | 0.48 [0.001, 0.86] | $d^{-1}$ |
| $\boldsymbol{r}$ | $C$ replication rate | 3.8 [2.7, 5.3] | 3.8 [2.7, 5.3] | 3.7 [2.6, 5.3] | 2.5 [1.7, 3.6] | 2.5 [1.7, 3.6] | 2.6 [1.8, 3.7] | $d^{-1}$ |
| $\boldsymbol{\alpha}$ | $R$ replication rate | 32.7 [25.0, 42.9] | 32.8 [25.1, 43.0] | 32.6 [24.7, 42.1] | 39.1 [29.9, 51.4] | 39.1 [29.9, 54.1] | 35.3 [27.1, 46.1] | $d^{-1}$ |
| $\boldsymbol{\mu}_{\boldsymbol{T}}$ | Cytoplasmic RNA degradation rate | 22.4 [16.2. 29.4] | 22.9 [16.4, 26.8] | 21.5 [16.2, 29.4] | 24.5 [17.2, 27.4] | 24.5 [17.2, 27.4] | 24.0 [16.8, 34.9] | $d^{-1}$ |
| $\boldsymbol{\mu}_{\boldsymbol{R}}$ | $R$ and $C$  degradation rates | 0.42 [0.15, 0.77] | 0.43 [0.15, 0.77] | 0.04 [0.01, 0.75] | 0 | 0 | 0 | $d^{-1}$ |
