# Supplementary material for: Mathematical modeling of hepatitis C RNA replication, exosome secretion and virus release
Source: PLoS Comput Biol. 2020 Nov 5;16(11):e1008421. doi: 10.1371/journal.pcbi.1008421 (PMC7671504; doi:10.1371/journal.pcbi.1008421)
Supplement: S2 Table — Parameter values of the best-fit models with a delayed ramp-up secretion (type 1 models: SMT1 = R1 and SMT1≠R1), simple time delayed secretion (type 2 models: SMT2 = R2 and SMT2≠R2), and an exponential decreasing secretion (type 3 models: SMT3 = R3 and SMT3≠R3). Note that for models where HCV RNA is secreted from both routes (the site of translation and the RC) we discriminate between two different cases: (i) secretion specific model parameters are the same for both secretion routes, i.e. τT = τR and ρT = ρR (SMTi = Ri with i = 1,2,3), or (ii) secretion specific model parameters are individual for both secretion routes, i.e. τT≠τR and ρT≠ρR (SMTi≠Ri with i = 1,2,3). Parameter values in [] show 95% confidence intervals, while values marked with * were kept fixed throughout the profile likelihood estimation. (DOCX) [file pcbi.1008421.s012.docx]

| Parameter | Description | SM_T1=R1_ | SM_T2=R2_ | SM_T3=R3_ | SM_T1≠R1_ | SM_T2≠R2_ | SM_T3≠R3_ | Unit |
| --- | --- | --- | --- | --- | --- | --- | --- | --- |
| $\boldsymbol{AIC}$ |  | 113.5 | **110.7** | 113.0 | 115.3 | **114.4** | 116.8 |  |
| $\boldsymbol{\rho}_{\boldsymbol{T}}$ | $S$ secretion rate | 0.028 [0.023, 0.034] | 0.026 [0.02, 0.034] | 0.027 [0.021, 0.034] | 0.036 [0.012, 1000] | 0.045 [0.02, 0.1] | 0.036 [0.015, 0.08] | $d^{-1}$ |
| $\boldsymbol{\rho}_{\boldsymbol{R}}$ | $S$ secretion rate |  |  |  | 0.026 [0, 0.033] | 0.026 [0  02, 0.03] | 0.026 [0.02, 0.034] | $d^{-1}$ |
| $\boldsymbol{\tau}_{\boldsymbol{\rho}_{\boldsymbol{T}}}$ | $S$ secretion delay | 0.01 [0.01, 0.26] | 0.01 [0.01, 0.04] | 0.01 [0.01, 0.04] | 0.005 [0.01, 0.5] | 0.02 [0.01, 5] | 0.01 [0.01, 5] | $d$ |
| $\boldsymbol{\tau}_{\boldsymbol{\rho}_{\boldsymbol{R}}}$ | $S$ secretion delay |  |  |  | 0.34 [0.01, 0.9] | 0.36 [0.01, 0.73] | 0.16 [0.01, 0.77] | $d$ |
| $\boldsymbol{k}_{\boldsymbol{\rho}_{\boldsymbol{T}}}\boldsymbol{=}\boldsymbol{k}_{\boldsymbol{\rho}_{\boldsymbol{R}}}$ | $S$ secretion rate parameter | 100 * | - | 0.01 * | 0.1 * | - | 0.01 * | $d^{-1}$ |
| $\boldsymbol{T}_{\boldsymbol{0}}$ | Initial number of RNAs transfected into the cell | 250 [143, 250] | 239 [123, 450] | 233 [121, 440] | 191 [150, 250] | 191 [150, 250] | 190 [150, 250] | molecules/cell |
| $\boldsymbol{C}_{\boldsymbol{max}}$ | Maximal number of $C$ | 31.9 [26.8, 38.1] | 32.1 [27.0, 38.4] | 32.2 [27.0, 38.4] | 32.3 [27.1, 38.5] | 32.3 [27.1, 38.5] | 32.3 [27.1, 38.5] | molecules/cell |
| $\boldsymbol{\sigma}$ | Rate of transfer of $T$ to the RC | 0.006 [0.0033, 0.01] | 0.006 [0.003, 0.01] | 0.006 [0.003, 0.01] | 0.007 [0.002, 0.02] | 0.007 [0.002, 0.02] | 0.007 [0.002, 0.02] | $d^{-1}$ |
| $\boldsymbol{\theta}$ | Rate of transfer of $R$to the cytoplasm | 0.42 [0.001, 0.77] | 0.42 [0.001, 0.77] | 0.42 [0.001, 0.77] | 0.42 [0.001, 0.78] | 0.42 [0.001, 0.78] | 0.42 [0.001, 0.77] | $d^{-1}$ |
| $\boldsymbol{r}$ | $C$ replication rate | 3.8 [2.7, 5.4] | 3.7 [2.6, 5.3] | 3.7 [2.6, 5.3] | 3.7 [2.6, 5.3] | 3.7 [2.6, 5.3] | 3.7 [2.6, 5.3] | $d^{-1}$ |
| $\boldsymbol{\alpha}$ | $R$ replication rate | 31.5 [24.3, 40.9] | 32.0 [24.5, 41.8] | 31.9 [24.5, 41.8] | 32.0 [24.5, 41.8] | 32.0 [24.5, 41.8] | 32.0 [24.5, 41.7] | $d^{-1}$ |
| $\boldsymbol{\mu}_{\boldsymbol{T}}$ | Cytoplasmic RNA degradation rate | 23.2 [19.5, 25.4] | 23.2 [18.8, 27.9] | 23.0 [18.7, 27.7] | 22.0 [15.5, 29.2] | 22.0 [15.6, 29.2] | 22.0 [15.6, 29.2] | $d^{-1}$ |
| $\boldsymbol{\mu}_{\boldsymbol{R}}$ | $R$ and $C$  degradation rates | 0 | 0 | 0 | 0 | 0 | 0 | $d^{-1}$ |
