# Supplementary material for: Mathematical modeling of hepatitis C RNA replication, exosome secretion and virus release
Source: PLoS Comput Biol. 2020 Nov 5;16(11):e1008421. doi: 10.1371/journal.pcbi.1008421 (PMC7671504; doi:10.1371/journal.pcbi.1008421)
Supplement: S3 Table — AICs, number of estimated model parameters (#P), time delay parameters (kρi,τρi,ρi) values of the best-fit models that take into account the secretion of (-)RNA and were fitted to measurements of plus-strand RNA, minus-strand RNA, and secreted HCV RNA in Keum et al. [35]. The model with the lowest AIC is highlighted in beige and the lowest AIC for each model is shown in bold. (DOCX) [file pcbi.1008421.s013.docx]

| Model | Secretion route | Time delay function | #P | $\boldsymbol{k}_{\boldsymbol{\rho}_{\boldsymbol{i}}}$ | $\boldsymbol{\tau}_{\boldsymbol{p}_{\boldsymbol{i}}}$ | $\boldsymbol{\rho}_{\boldsymbol{i}}$ | AIC |
| --- | --- | --- | --- | --- | --- | --- | --- |
| SM_C1_ | (-)RNA secretion from the RC ($C$) | Delay then ramp-up (Eq. 2) | 11 | $k_{C}=100 d^{-1}$ | $\tau_{C}=0.01 d$ | $\rho_{C}=0.6 d^{-1}$ | 192.9 |
| SM_C2_ |  | Simple delay (Eq. 3) | 10 | - | $\tau_{C}=0.01 d$ | $\rho_{C}=0.6 d^{-1}$ | **190.0** |
| SM_C3_ |  | Delayed exponential decrease (Eq. 4) | 11 | $k_{C}=0.01 d^{-1}$ | $\tau_{C}=0.01 d$ | $\rho_{C}=0.6 d^{-1}$ | 193.7 |
| SM_T1=C1_ | (-)RNA and (+)RNA secretion from the RC ($C$) and the site of translation ($T$) with $\rho_{T}= \rho_{C}$ and $\tau_{T}= \tau_{C}$ | Delay then ramp-up (Eq. 2) | 11 | $k_{C}=k_{T}=0.01 d^{-1}$ | $\tau_{C}=\tau_{T}=0.1 d$ | $\rho_{C}=\rho_{T}=27 d^{-1}$ | **115.7** |
| SM_T2=C2_ |  | Simple delay (Eq. 3) | 10 | - | $\tau_{C}=\tau_{T}=0.1 d$ | $\rho_{C}=\rho_{T}=0.26 d^{-1}$ | 133.1 |
| SM_T3=C3_ |  | Delayed exponential decrease (Eq. 4) | 11 | $k_{C}=k_{T}=0.01 d^{-1}$ | $\tau_{C}=\tau_{T}=0.1 d$ | $\rho_{C}=\rho_{T}=0.26 d^{-1}$ | 136.1 |
| SM_T1≠C1_ | (-)RNA and (+)RNA secretion from the RC ($C$) and the site of translation ($T$) with $\rho_{T}\neq\rho_{C}$ and $\tau_{T}\neq\tau_{C}$ | Delay then ramp-up (Eq. 2) | 13 | $k_{C}=k_{T}=10 d^{-1}$ | $\tau_{C}=3 d$  $\tau_{T}=0.3 d$ | $\rho_{C}=2 d^{-1}$  $\rho_{T}=1000 d^{-1}$ | **105.5** |
| SM_T2≠C2_ |  | Simple delay (Eq. 3) | 12 | - | $\tau_{C}=0.3 d$  $\tau_{T}=0.2 d$ | $\rho_{C}=0.05 d^{-1}$  $\rho_{T}=1.1 d^{-1}$ | 113.8 |
| SM_T3≠C3_ |  | Delayed exponential decrease (Eq. 4) | 13 | $k_{C}=k_{T}=0.01 d^{-1}$ | $\tau_{C}=0.02 d$  $\tau_{T}=0.17 d$ | $\rho_{C}=0.05 d^{-1}$  $\rho_{T}=1.2 d^{-1}$ | 116.8 |
| SM_T1=R1=C1_ | (-)RNA and (+)RNA secretion from the RC ($C,R$) and the site of translation ($T$) with $\rho_{T}= \rho_{R}=\rho_{C}$ and $\tau_{T}=\tau_{R}=\tau_{C}$ | Delay then ramp-up (Eq. 2) | 11 | $k_{C}=k_{T}=k_{R}=100 d^{-1}$ | $\tau_{C}=\tau_{T}=\tau_{R}=0.1 d$ | $\rho_{C}=\rho_{T}=\rho_{R}=0.026 d^{-1}$ | 113.1 |
| SM_T2=R2=C2_ |  | Simple delay (Eq. 3) | 10 | - | $\tau_{C}=\tau_{T}=\tau_{R}=0.1 d$ | $\rho_{C}=\rho_{T}=\rho_{R}=0.025 d^{-1}$ | **110.5** |
| SM_T3=R3=C1_ |  | Delayed exponential decrease (Eq. 4) | 11 | $k_{C}=k_{T}=k_{R}=0.01 d^{-1}$ | $\tau_{C}=\tau_{T}=\tau_{R}=0.1 d$ | $\rho_{C}=\rho_{T}=\rho_{R}=0.025 d^{-1}$ | 112.8 |
| SM_T1≠R1≠C1_ | (-)RNA and (+)RNA secretion from the RC ($C,R$) and the site of translation ($T$) with $\rho_{T}\neq\rho_{R}\neq\rho_{C}$ and $\tau_{T}\neq\tau_{R}\neq\tau_{C}$ | Delay then ramp-up (Eq. 2) | 15 | $k_{C}=k_{T}=k_{R}=5 d^{-1}$ | $\tau_{C}=0.01 d$  $\tau_{T}=0.06 d$  $\tau_{R}=2.7 d$ | $\rho_{C}=0.14 d^{-1}$  $\rho_{T}=0.44 d^{-1}$  $\rho_{R}=0.05 d^{-1}$ | **76.9** |
| SM_T2≠R2≠C2_ |  | Simple delay (Eq. 3) | 14 | - | $\tau_{C}=0.2 d$  $\tau_{T}=0.2 d$  $\tau_{R}=3 d$ | $\rho_{C}=0.1 d^{-1}$  $\rho_{T}=0.7 d^{-1}$  $\rho_{R}=0.04 d^{-1}$ | 89.7 |
| SM_T3≠R3≠C3_ |  | Delayed exponential decrease (Eq. 4) | 15 | $k_{C}=k_{T}=k_{R}=0.05 d^{-1}$ | $\tau_{C}=0.1 d$  $\tau_{T}=0.03 d$  $\tau_{R}=2.7 d$ | $\rho_{C}=0.3 d^{-1}$  $\rho_{T}=0.04 d^{-1}$  $\rho_{R}=0.05 d^{-1}$ | 102.7 |
| SM_T1≠R1≠C1_ | (-)RNA and (+)RNA secretion from the RC ($C,R$) and the site of translation ($T$) with $\rho_{T}\neq\rho_{R}\neq\rho_{C}$ and $\tau_{T}\neq\tau_{R}\neq\tau_{C}$ and $k_{T}\neq k_{R}\neq k_{C}$ | Delay then ramp-up (Eq. 2) | 17 | $k_{C}=100 d^{-1}$  $k_{T}=100 d^{-1}$  $k_{R}=1 d^{-1}$ | $\tau_{C}=0.5 d$  $\tau_{T}=0.1 d$  $\tau_{R}=2.5 d$ | $\rho_{C}=0.14 d^{-1}$  $\rho_{T}=0.3 d^{-1}$  $\rho_{R}=0.07 d^{-1}$ | **78.8** |
