# Supplementary material for: Mathematical modeling of hepatitis C RNA replication, exosome secretion and virus release
Source: PLoS Comput Biol. 2020 Nov 5;16(11):e1008421. doi: 10.1371/journal.pcbi.1008421 (PMC7671504; doi:10.1371/journal.pcbi.1008421)
Supplement: S4 Table — Parameter values of the best-fit model that consider the secretion of HCV (+)RNA and (-)RNA (S2 and S4 Figs, S3 Table) with a delayed ramp-up secretion. Note that every secretion route is individual and hence, kT≠kR≠kC, τT≠τR≠τC, and ρT≠ρR≠ρC. Parameter values in [] show 95% confidence intervals, while values marked with * were kept fixed throughout the profile likelihood estimation. The degradation rate of HCV RNA within the RC was set to μR = 0 (see main text). (DOCX) [file pcbi.1008421.s014.docx]

| Parameter | Description | SM_T1≠R1≠M1_ | Unit |
| --- | --- | --- | --- |
| $\boldsymbol{AIC}$ |  | **78.8** |  |
| $\boldsymbol{\rho}_{\boldsymbol{T}}$ | $S$ secretion rate from $T$ | 0.32 [1E-5, 1000] | $d^{-1}$ |
| $\boldsymbol{\rho}_{\boldsymbol{R}}$ | $S$ secretion rate from $R$ | 0.07 [0.055, 0.097] | $d^{-1}$ |
| $\boldsymbol{\rho}_{\boldsymbol{C}}$ | $S$ secretion rate from $C$ | 0.14 [0.10, 0.19] | $d^{-1}$ |
| $\boldsymbol{\tau}_{\boldsymbol{\rho}_{\boldsymbol{T}}}$ | $S$ secretion delay from $T$ | 0.1 [0.01, 3] | $d$ |
| $\boldsymbol{\tau}_{\boldsymbol{\rho}_{\boldsymbol{R}}}$ | $S$ secretion delay from $R$ | 2.5 [2.4, 2.6] | $d$ |
| $\boldsymbol{\tau}_{\boldsymbol{\rho}_{\boldsymbol{C}}}$ | $S$ secretion delay from $C$ | 0.5 [0.5, 0.56] | $d$ |
| $\boldsymbol{k}_{\boldsymbol{\rho}_{\boldsymbol{T}}}$ | $S$ secretion rate parameter from $T$ | 100 * | $d^{-1}$ |
| $\boldsymbol{k}_{\boldsymbol{\rho}_{\boldsymbol{R}}}$ | $S$ secretion rate parameter from $R$ | 1 * | $d^{-1}$ |
| $\boldsymbol{k}_{\boldsymbol{\rho}_{\boldsymbol{C}}}$ | $S$ secretion rate parameter from $C$ | 100 * | $d^{-1}$ |
| $\boldsymbol{T}_{\boldsymbol{0}}$ | Initial number of HCV RNAs | 192 [150, 250] | molecules/cell |
| $\boldsymbol{C}_{\boldsymbol{max}}$ | Maximal number of $C$ | 32.1 [27.4, 37.8] | molecules/cell |
| $\boldsymbol{\sigma}$ | Rate of transfer of $T$ to the RC | 0.008 [1E-5, 1000] | $d^{-1}$ |
| $\boldsymbol{\theta}$ | Rate of transfer of $R$to the cytoplasm | 0.6 [1E-5, 0.96] | $d^{-1}$ |
| $\boldsymbol{r}$ | $C$ replication rate | 3.4 [1E-5, 1000] | $d^{-1}$ |
| $\boldsymbol{\alpha}$ | $R$ replication rate | 35.2 [1E-5, 1000] | $d^{-1}$ |
| $\boldsymbol{\mu}_{\boldsymbol{T}}$ | Cytoplasmic RNA degradation rate | 22.1 [20, 25] | $d^{-1}$ |
| $\boldsymbol{\mu}_{\boldsymbol{R}}$ | $R$ and $C$  degradation rates | 0 | $d^{-1}$ |
