# Supplementary material for: Mathematical modeling of hepatitis C RNA replication, exosome secretion and virus release
Source: PLoS Comput Biol. 2020 Nov 5;16(11):e1008421. doi: 10.1371/journal.pcbi.1008421 (PMC7671504; doi:10.1371/journal.pcbi.1008421)
Supplement: S1 Text — (DOCX) [file pcbi.1008421.s015.docx]

**S1 Text: Minus-strand and plus-strand HCV RNA as secretion sources**

To determine whether minus-strand RNA [(-)RNA] serves as an additional source to the plus-strand RNA [(+)RNA] secretion, we extended our intracellular HCV replication and secretion model by the (-)RNA secretion rate $\rho_{C}$ (Eq. S1 and S6 Fig). Thus, in addition to (+)RNA secretion from the site of translation ($\rho_{T}$) and/or the RC ($\rho_{R}$), we studied the (-)RNA secretion of (-)RNA (or dsRNA) from the RC ($\rho_{C}$), which is given by the following set of ODEs:

$$\begin{aligned} \frac{d}{dt}T&=\theta R-\left( \sigma+\rho_{T}\left( t \right)+\mu_{T} \right)T, \\ \frac{d}{dt}R&=\alpha C+\sigma T-\left( \theta+\rho_{R}\left( t \right)+\mu_{R} \right)R, \\ \frac{d}{dt}C=r\left( 1-\frac{C}{C_{max}} \right)R-{(\boldsymbol{\rho}_{\boldsymbol{C}}\left( \boldsymbol{t} \right)+\mu}_{R})C, \\ \frac{d}{dt}S=\rho_{T}\left( t \right)T+\rho_{R}\left( t \right)R\boldsymbol{+}\boldsymbol{\rho}_{\boldsymbol{C}}\left( \boldsymbol{t} \right)\boldsymbol{C}. \end{aligned}\left( S1 \right)$$

With this extended model, we studied three different HCV RNA secretion scenarios: (i) (-)RNA secretion from the RC (SM_C_), (ii) (+)RNA secretion from the site of translation and (-)RNA from the RC (SM_TC_), as well as (iii) (+)RNA secretion from the site of translation and (+)RNA and (-)RNA from the RC (SM_TRC_). For (ii) and (iii), we discriminate further whether the HCV RNA secretion is equal for each secretion route or individual; i.e. ($\tau_{T}=\tau_{R}=\tau_{C}$, $\rho_{T}=\rho_{R}=\rho_{C}$) or ($\tau_{T}\neq\tau_{R}\neq\tau_{C}$, $\rho_{T}\neq\rho_{R}\neq\rho_{C}$), respectively. Similarly to the models which account only for (+)RNA as HCV RNA secretion sources, we fitted the additional models to the Keum et al. [1] data set and studied a delayed ramp-up (type 1 models), a simple time delayed secretion (type 2 models), and an exponentially decreasing secretion (type 3 models). Similarly to the model neglecting HCV (-)RNA (or dsRNA) secretion the type 1 (ramp up) and 3 (exponentially decreasing) models approximate for type 2 (simple time delayed secretion) models for high and low secretion rate parameters ($k_{\rho_{i}}$), respectively (S7 Fig).

However, we observed the overall best model fit with a delayed ramp-up HCV RNA secretion (type 1 model) individually from all three sources (S4 Table). We further studied that model concerning different secretion rate parameters $k_{p_{i}}$ with $k_{T}\neq k_{R}\neq k_{C}$. Comparing the best fit model and the model with individual $k_{p_{i}}$ parameters showed a 1.9 higher AIC suggesting no preference for either model. Studying individual HCV (+) and (-)RNA secretion routes showed that only (+)RNA from the RC follows a time delayed ramp-up (type 1), while (+)RNA from the site of translation and (-)RNA from the RC approximate for a simple time delay (type 2) with $k_{\rho_{T}}=k_{\rho_{C}}=100 d^{-1}$ (S3 and S4 Tables, S8 Fig). Furthermore, the estimated time delays show that (+)RNA from the site of translation is secreted after 2.4 hours, (-)RNA from the RC after 12 hours and (+)RNA from the RC is secreted after 2.5 days.

The best-fit model with individual secretion rate parameters ($k_{\rho_{i}}$) is shown in S8 Fig, the corresponding model parameters and 95% confidence intervals are listed in S4 Table (the identifiability profiles of model parameters are shown in S9 Fig).

[1] Keum SJ, Park SM, Park JH, et al. The specific infectivity of hepatitis C virus changes through its life cycle. *Virology* 2012; 433: 462–470.
