# Supplementary material for: Mathematical modeling of hepatitis C RNA replication, exosome secretion and virus release
Source: PLoS Comput Biol. 2020 Nov 5;16(11):e1008421. doi: 10.1371/journal.pcbi.1008421 (PMC7671504; doi:10.1371/journal.pcbi.1008421)
Supplement: S2 Text — (DOCX) [file pcbi.1008421.s016.docx]

**S2 Text: Sensitivity Analysis of the best fit model (CM_4_)**

A sensitivity analysis has been performed for plus- and minus-strand RNA, secreted RNA, as well as intracellular and extracellular infectious virus (1 d pi and 3 d pi; see main text for details). Note that since virus assembly and release is delayed, we performed sensitivity analysis for intracellular and extracellular infectious virus only for day 3 post infection (S10 Fig).

For plus- and minus-strand HCV RNA, RNA synthesis within the RC ($r$, $\alpha$, and $C_{max}$) as well as the transfer of HCV RNA from the site of translation into the RC ($\sigma$) represented the most sensitive processes in the viral lifecycle. Especially, processes within the RC showed the highest significant sensitivity early and late in infection (S10A-D Fig).

For secreted HCV RNA, the rate of secretion from the site of translation ($\rho_{T}$) showed a high sensitivity early and late post-infection (1 d pi, S10E and S10F Fig). However, later in infection (3 d pi), secreted HCV RNA seems to be influenced by HCV assembly and release processes ($f(t)$ and $\nu$), which seems to be competitive processes.

For intracellular and extracellular virus (S10G and S10H Figs), HCV RNA synthesis within the RC showed high sensitivity ($\alpha$), as well as virus assembly and release processes ($f\left( t \right)$ and $\nu$). Interestingly, for intracellular virus, changes in the HCV RNA secretion rate ($\rho_{T}$ or $\rho_{R}$) show significant sensitivities and thus might represent competitive processes for virus assembly and release. However, for extracellular infectious virus secretion processes seem to have no effect. Taken together, for all studied HCV species, HCV RNA synthesis within the RC, HCV RNA secretion and virus assembly and release represented the most sensitive processes in the viral lifecycle.
